# From bowls to pots: The dairying revolution in Northwest Turkey, a view from Barcın Höyük, 6600 to 6000 BCE

**DOI:** 10.1371/journal.pone.0302788

**Published:** 2024-05-09

**Authors:** Hadi Özbal, Adria Breu, Laurens Thissen, Fokke Gerritsen, Elisha van den Bos, Alfred Galik, Turhan Doğan, Muhiddin Çergel, Adnan Şimşek, Ayla Türkekul, Rana Özbal

**Affiliations:** 1 Boğaziçi University, Istanbul, Turkey; 2 Koç University, Istanbul, Turkey; 3 Thissen Archaeological Ceramics Bureau, Bureau, The Netherlands; 4 Netherlands Institute in Turkey, Istanbul, Turkey and Leiden University, Leiden, The Netherlands; 5 Barcın Höyük Research Project, Istanbul, Turkey; 6 Austrian Academy of Sciences, Vienna, Austria; 7 Tübitak MAM Marmara Research Center, Gebze, Turkey; 8 Tübitak UME National Metrology Institute, Gebze, Turkey; Israel Antiquities Authority, ISRAEL

## Abstract

Research has identified Northwest Turkey as a key region for the development of dairying in the seventh millennium BCE, yet little is known about how this practice began or evolved there. This research studies Barcın Höyük, a site located in Bursa’s Yenişehir Valley, which ranges chronologically from 6600 BCE, when the first evidence of settled life appears in the Marmara Region, to 6000 BCE, when Neolithic habitation at the site ceases. Using pottery sherds diagnostic by vessel category and type, this paper aims at identifying which ones may have been primarily used to store, process, or consume dairy products. Organic residue analysis of selected samples helped address the process of adoption and intensification of milk processing in this region over time. The lipid residue data discussed in this paper derive from 143 isotopic results subsampled from 173 organic residues obtained from 805 Neolithic potsherds and suggest that bowls and four-lugged pots may have been preferred containers for processing milk. The discovery of abundant milk residues even among the earliest ceramics indicates that the pioneer farmers arrived in the region already with the knowhow of dairying and milk processing. In fact, these skills and the reliance on secondary products may have given them one of the necessary tools to successfully venture into the unfarmed lands of Northwest Anatolia in the first place.

## Introduction

The production and consumption of milk-based foods counts among the most important secondary innovations in the establishment of food producing economies [1–3]. While zooarchaeological studies based on culling patterns may place the incipient stages of dairying in the 8^th^ millennium BCE [4–6], methodological and statistical considerations and inadequacies in sample size imply that additional independent sources of data for dairying are necessary [7–9]. Alternatively, the detection of milk lipids as organic residues in archaeological pottery (based on the δ^13^C and Δ^13^C values of the two major alkanoic acids of milk fat) has, over the past few decades, allowed researchers to determine that ruminants were exploited for their milk just after their first domestication [10–12], much sooner than what was suggested by the earliest human aDNA evidence of the lactase persistence genes [13,14].

**Fig 1:**
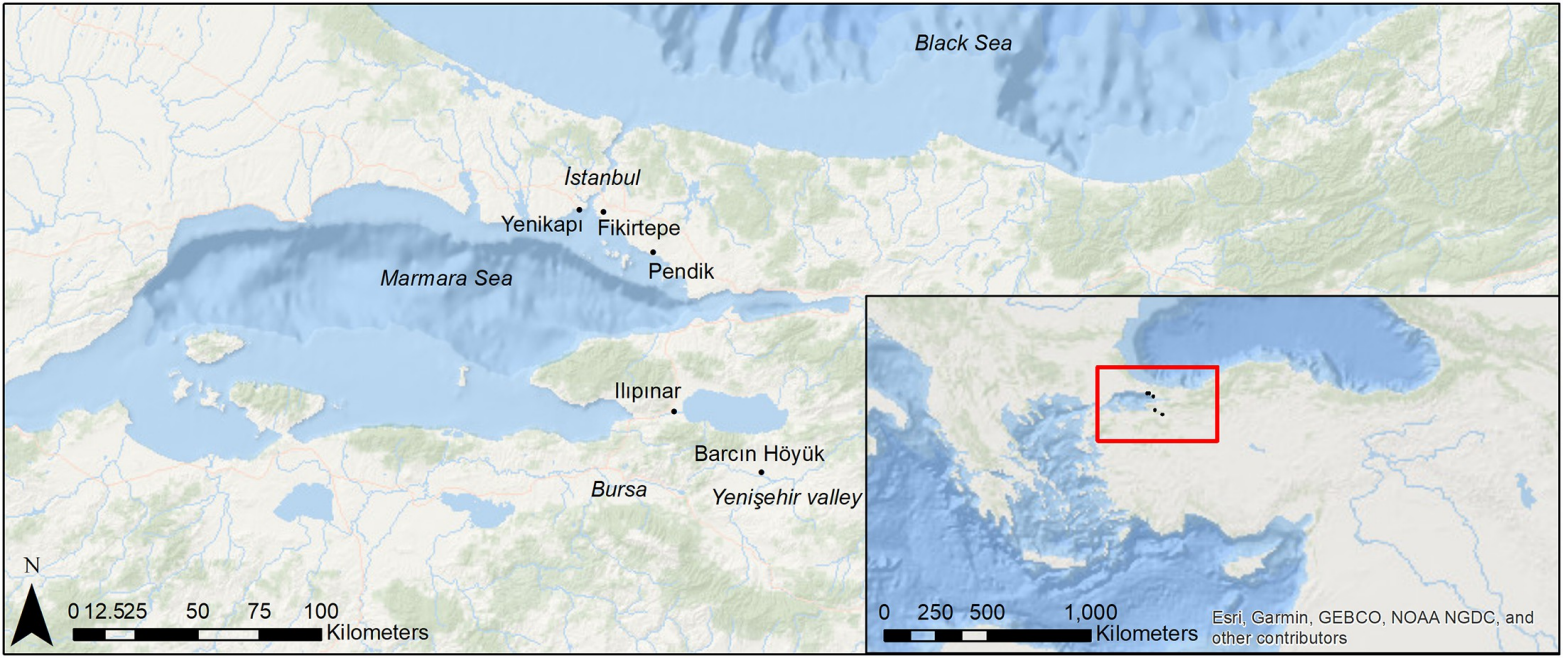
Map of northwestern Turkey with the sites mentioned in the text.

Across prehistoric Europe and Southwest Asia, dairying would be practiced in different scales and intensities through time. While it was shown to be prevalent in pottery residues from the sixth millennium northern Balkans and the Carpathian basin [15], and from fourth millennium Neolithic Britain and Ireland, it was rare in early Neolithic settlements in the North European plain and in the Mediterranean [10,16–23]. However, in a groundbreaking study comparing the fifth, sixth and seventh millennium BCE sites from northern Mesopotamia, parts of Anatolia and southeastern Europe, a surprisingly high ratio of dairy residues was discovered in the region surrounding the Marmara Sea in northwestern Turkey [10] (Fig 1), thus highlighting the importance of this territory, as it may have hosted the first comprehensive adoption of dairying. Since then and despite the inclusion of Anatolia in recent Mediterranean and European-wide meta-studies [13,24,25], only advances on a single site [26,27] have been made in trying to understand how this process began or evolved in the peninsula.

A neolithic lifestyle, agriculture and animal husbandry are necessary for dairying economies to emerge. Evidence for this way of living is known from Central Anatolia from the ninth millennium onwards, but it did not spread westwards towards the Sea of Marmara until the first half of the seventh millennium [28–30]. Although recent research has isolated 6500 BCE as an approximate date for the appearance of milk lipids in northwestern Turkey [10], a detailed study focusing on this phenomenon using a well-sequenced multiphase site is imperative for a thorough understanding of the emergence and evolution of dairying in this region in the seventh millennium. Furthermore, a knowledge gap on the vessel types most frequently involved in the storage, transport, transformation, and consumption of dairy products has severely limited the capacity of researchers to explore the social and economic implications of this product. Given the apparent dissociation between the intensity of dairying practices and the emergence of the lactase persistence gene [13], exploring cultural factors has become indispensable to understand the reasons behind the initial success of dairying among human populations.

Dating between 6600 and 6000 cal. BCE, the site of Barcın Höyük, located in the Yenişehir Valley in northwestern Turkey (Fig 1), provides an excellent dataset to study the emergence of dairying economies. The site uses a single context recording system, resulting in levels backed up by over 80 radiocarbon dates, making each context secure [31,32]. The earliest levels of the site represent a time when the initial Neolithic pioneers arrived in this region and its uninterrupted Neolithic chronological sequence enables teasing apart, stage by stage, the incipient appearance of dairying. The study of more than 800 Neolithic sherds presented here make Barcın Höyük the most extensively analyzed single site to date for organic residue analyses across Eurasia and Africa, providing us with the potential to offer a fresh look at the question of the intensity, scale and character of dairying through several centuries, from the start of the Neolithic lifeways in the region, until the turn of the millennium, when the settlement was abandoned [30,32–35]. Previous research from Barcın Höyük has shown that dairy products were an important nutritional component for the earliest colonizing pioneers [20,36–39]. However, to move beyond pinpointing the earliest appearance of milk, heading the call by Greenfield and Arnold [40], this paper will aim at assessing changes in dairy production and consumption practices by identifying the roles of different Neolithic vessel types, as this remains an important factor in understanding changes in Neolithic subsistence patterns.

## Materials and methods

### Background on the site and the ceramic assemblage studied

**Fig 2:**
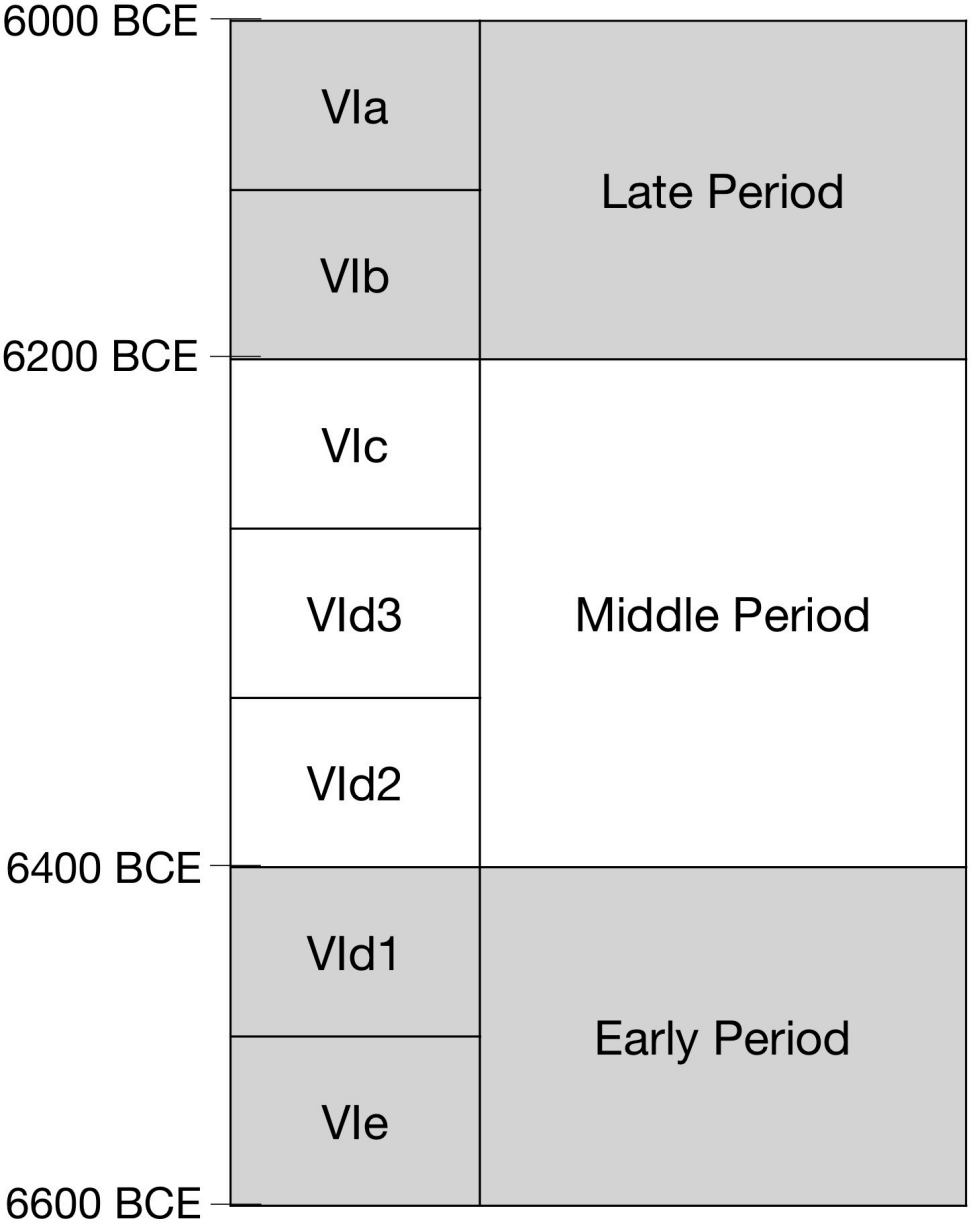
Occupation sequence at Barcın Höyük with stratigraphic phases, analytical periods and generalized absolute dates.

Based mainly on stratigraphic analyses but also on the correlation of the gradual changes in ceramic features including finish, shape, form and temper, the ceramics from the Neolithic levels of Barcın Höyük have been assigned to one of seven phases from VIe-VIa (Fig 2). In this paper, however, the 805 vessels tested for the presence of lipid organic residues originating from these seven phases have been grouped into three larger analytical Periods, each covering approximately a 200-year timeframe, ensuring a thorough coverage of the most common vessel types per phase. Thus, phases VIe-VId1 are combined in an Early Period (ca. 6600–6400 cal. BCE) with 262 samples, phases VId2-VIc are combined in a Middle Period (ca. 6400–6200 cal. BCE) with 233 samples, and phases VIb-VIa are combined in a Late Period, (ca. 6200–6000 cal. BCE) from which 310 samples were collected (Fig 2). These Periods, created to enlarge sample sizes, present robust analytical categories representing the evolution of pottery forms, shapes and styles across time.

**Fig 3:**
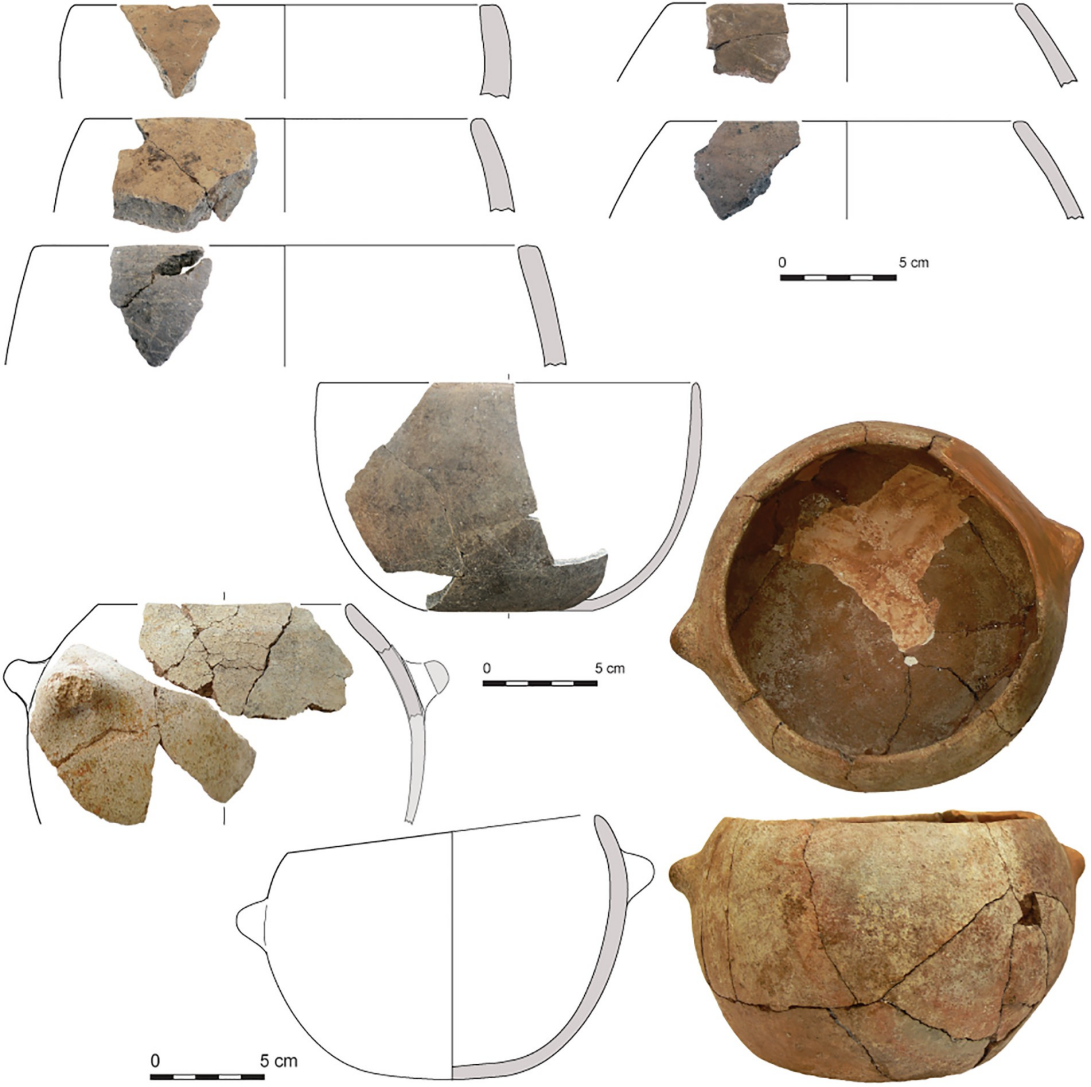
Drawings and integrated photos of VId1 or VIe (Early Period) pottery from Barcın Höyük showing VIe thick-walled hole-mouth pots on the upper half as well as a bowl in the center and knobbed and pierced pots from VId1 on the lower half.

**Fig 4:**
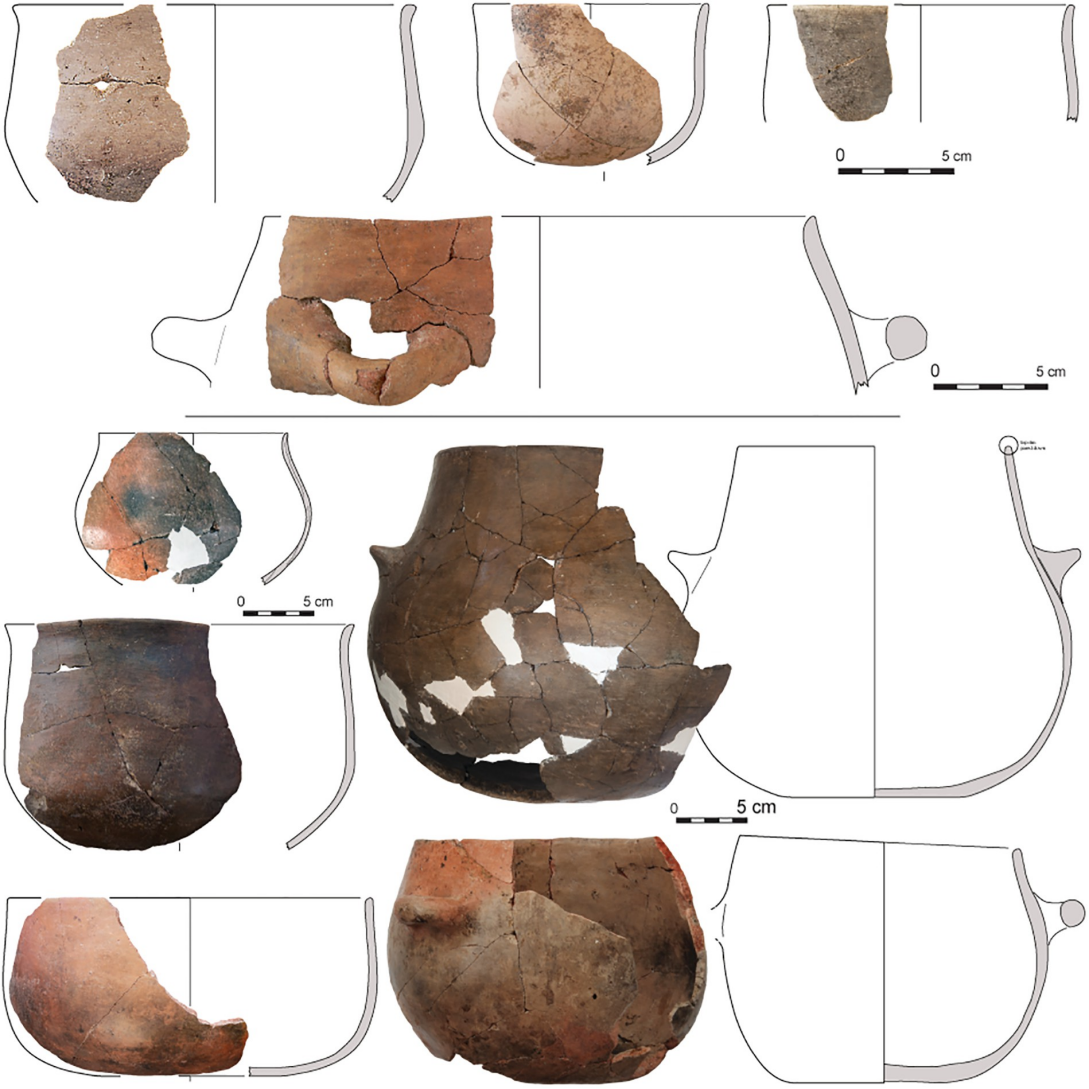
Drawings and integrated photos of VId3, VId2 or VIc (Middle Period) pottery from Barcın Höyük showing bowls at the top and lower left and two-lugged pots in the lower right.

**Fig 5:**
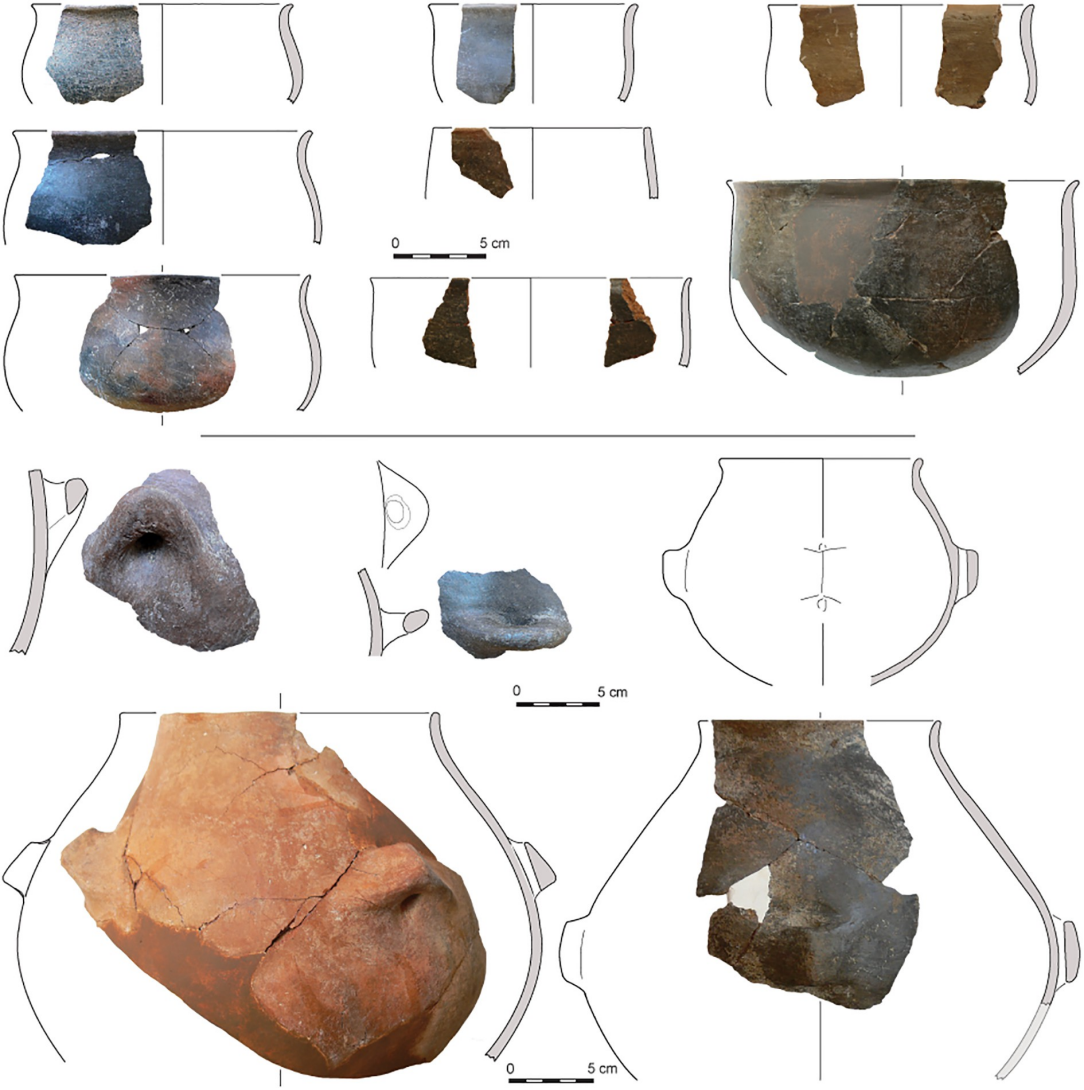
Drawings and integrated photos of VIa or VIb (Late Period) pottery from Barcın Höyük showing examples of S-shaped straight-sided bowls on the upper half and four-lugged suspension pots in the lower half.

The proposed division in ceramics is in agreement with general trends in the development of architecture at Barcın Höyük. Typical of the Early Period and Middle Period are mud-and-wood structures with east-west linearly arranged houses flanking large courtyards. By the Late Period, the settlement shows a new spatial arrangement with more free-standing houses built in former courtyards. Ceramics also show gradual yet distinct changes through time. It is important to emphasize that pottery was rare in the earliest stages of the Early Period, corresponding to Barcın VIe [33]. The vessels produced in this phase using micaceous and schist-tempered clays tended to be lightly burnished well-made wares. They were thick-walled and usually had holemouth shapes. When present, handles were often knob-like. Large quantities of fire-cracked stones suggest multiple ways of preparing food next to the use of pots for cooking [20,33]. It would be safe to say that the VIe ceramic assemblage, dating to the end of the first half of the seventh millennium BC, is among the earliest in the region. Slowly, by VId1, in the later stages of the Early Period, potters started making open and closed thinner-walled vessels and gradually finely-crushed calcite became the preferred temper, enabling possibly better thermoregulation over schist tempers. By the Middle Period, starting already by phase VId3 and continuing into the later phases of the Neolithic, the tempering showed a gradual shift from calcite to combinations of calcite together with schist, quartz and feldspars. Vessel walls, which range around 5–6 mm in phase VIc, gradually became thicker as the effort put into vessel production decreased towards the end of the seventh millennium BCE [22,23]. The uppermost Neolithic levels, corresponding to the Late Period, also present new characteristics, including dark colors and S-profiles, but are distinguished by the development of easily suspendable pots with four lugs (Figs 3–5).

**Fig 6:**
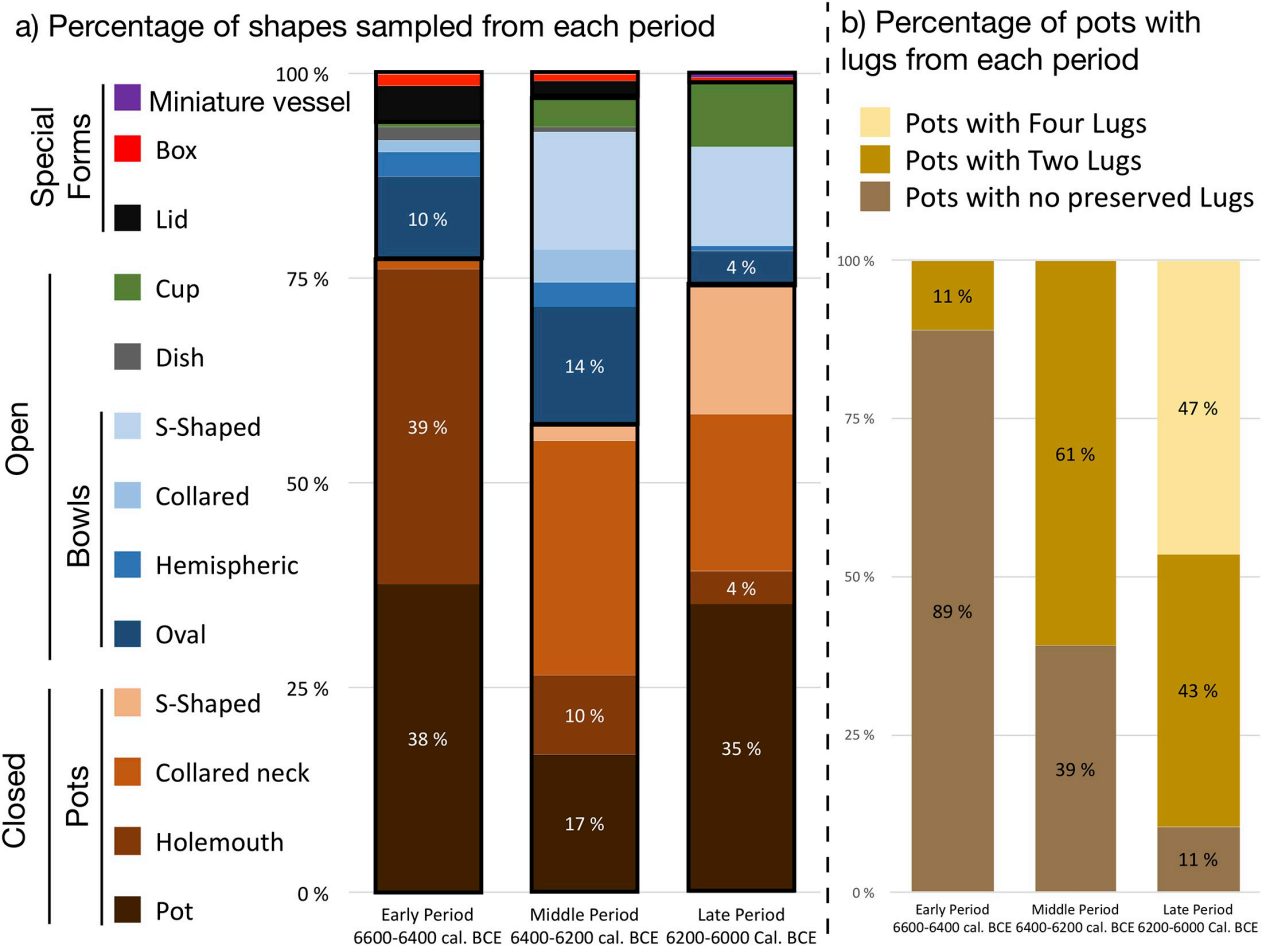
Typological composition of the studied assemblage at Barcın Höyük.

The study of lipid residues from pottery use at Barcın Höyük has had a long tradition [20,36–39]. This paper integrates new results from 344 unpublished sherds with an expanded reporting of 461 partially published samples [20,39]. The sampling strategy implemented for this overall research aimed at maximizing the study of pottery fragments indicative of the vessel category and type. Therefore, out of the 805 potsherds selected for organic residue analysis, the upper parts of the vessels were sampled in 67% of the cases, including zones near rims (N = 407, 51%), near handles or lugs (N = 123, 15%) and body fragments near necks or shoulders (N = 10, 1.3%). Samples were also taken from indeterminate body sherds (N = 169, 20%, especially for the earliest phases, where ceramics remains were rare) and fragments from the lower part of the vessel (12%) such as bases (N = 86, 10%) or walls near the base (N = 10, 2%). Overall, 77% of the samples corresponded to diagnostic sherds, which included 438 pots presenting different profile variations (holemouth, collared-neck, S-shaped, indeterminate), 133 bowls, including oval, hemispheric, collared and S-shaped types, 4 dishes, 28 cups, 12 lids, 6 boxes and one miniature vessel (see S1.1 in S1 File for additional details). Additionally, most of the sampled pots were fitted out with either two (N = 164, 37%) or four handles or lugs (N = 95, 22%) along their upper body. The number of vessels sampled per category and type were randomly collected on site and are thus assumed to be representative of their relative abundances in the Early, Middle and Late Periods [41] (Fig 6, S1 File and S1 Table).

### Lipid extraction protocols

The sampled area of all studied sherds was prepared by initially scraping their external, internal and side surfaces using a modelling drill with an abrasive bit. Subsequently, 1 or 2 grams of pottery were ground with pestle and mortar. The resultant powder was then subject to a chloroform/methanol (2/1, v/v) solvent extraction [42] in 681 cases or an H_2_SO_4_/MeOH (3% v/v) acid extraction (Acidified Methanol) [43,44] in a random selection of 113 samples (See S1 Table for details on the selection of extraction techniques). In 11 cases, solvent extractions were performed on samples that had already undergone the acid procedure (Table 1). For samples yielding positive results (TLE above 5 μg/g) with the chloroform/methanol procedure, further hydrolysis and methylation were conducted using a boron trifluoride procedure (see S1 File for procedure details) before compound-specific isotopic analyses. Blanks were run across samples and validated the absence of laboratory and cross-contamination.

**Table 1:** Extractions performed following the chloroform/methanol, the acidified methanol extraction or a combination of both for each analytical Period in Barcın Höyük.

Protocol	Early VId1, Vie		Middle VIc, VId3, VId2		Late VIa VIb		Total	
	N	%	n	%	n	%	N	%
Chloroform/Methanol	180	69	194	83	307	99	681	84
Acidified Methanol	73	28	37	16	3	1	113	14
Combined	9	3	2	1	0	0	11	2
Total	263		233		310		805	

Samples subjected to the chloroform/methanol extraction underwent analysis utilizing high-temperature-gas chromatography (HT-GC) [42], which was coupled to a flame ionization detector in a YL 6100C Gas Chromatograph. This process aimed to detect the presence of free fatty acids, monoacylglycerols, diacylglycerols, and triacylglycerols. Conversely, samples extracted via the Acidified Methanol procedure were analyzed using an ATI Unicam 610 Series Gas Chromatograph. To confirm and validate results obtained from this method, re-injections were performed using an Agilent 7820A Gas Chromatograph, with a specific focus on detecting free fatty acids. Additionally, GC-MS and GC-C-IRMS analyses were performed on samples presenting significant quantities of lipids and high peak level purity to identify major compounds and obtain compound-specific isotopic ratios (see S1 File for analytical details). Analyses were performed in Istanbul at Boğaziçi University’s Archaeometry laboratory in conjunction with the Archaeology Laboratory at Koç University and the TÜBİTAK MAM Marmara Research Center and the TÜBİTAK UME National Metrology Institute both located in Kocaeli, Turkey.

## Results

### Lipid preservation

Overall, 173 of 805 samples yielded interpretable lipids (21% of the studied assemblage) and 143 of them fulfilled the necessary criteria to be studied by isotopic analyses, which is in line with previous published reports from Barcın Höyük [20,36–39] (see S1 Table for additional details). The percentage of lipid-bearing samples (preservation rate) across the analytical Periods was relatively even. The Early Period contributed 52 samples (20%), the Middle Period added 51 samples (21%), and the Late Period contributed a slightly higher number, with 70 samples (23%). While some vessel categories and types (hemispheric bowls, collared bowls, dishes, lids, boxes and a miniature vessel) were not studied consistently enough to reliably evaluate their preservation rates, other types were extensively sampled, showing significant differences in the frequency in which they yielded a lipid signal (Tables 2 and 3). Whereas oval bowls yielded the highest reliable preservation rates in the Early and Middle Period (33% and 46% respectively), S-shaped pots from the Late Period had the highest recovery rate from this layer (44%). Pots with no preserved lugs showed a steady decline in the presence of lipids across the three phases (Table 3), but this was not the case for well-preserved two-lugged and four-lugged pots, which consistently present a lipid signal that aligns well with the general preservation rate of the site.

**Table 2:** Number of samples yielding lipids for each pottery shape and period, Pr: Preservation rate (%).

Pottery shapes		Early Period		Middle Period		Late Period		Total	
		n	Pr	N	Pr	n	Pr	n	Pr
Closed	Pot	18	27	8	27	14	14	40	21
	Holemouth pot	11	16	2	12	2	18	15	16
	Collared neck pot	0	0	4	8	7	13	11	11
	S-shaped pot	-		1	25*	20	45	21	44
Open	Oval bowl	6	33	12	50	5	42	23	41
	Hemispheric bowl	1	17*	1	17*	0	NC	2*	16*
	Collared bowl	0	0	0	0	-		0*	0*
	S-shaped bowl	-		8	33	10	31	18	32
	Dish	0	0	0	NC	-		0*	0*
	Cup	0	NC	3	50*	6	29	9	32
Special forms	Lid	1	13	0	0	-	0	1*	8*
	Box	0	0	0	NC	0	NC	0*	17*
	Miniature vessel	-		-		1	NC	1*	NC
Indeterminate		15	18	12	20	5	13	32	17
Total		52	20	51	21	70	23	173	21

* unreliable preservation rates due to the low amount of samples analyzed for the type.

**Table 3:** Number of positive samples associated with pots whose shapes include two or four lugs, or cases where these were not preserved.

Lugs in pots		Early Period		Middle Period		Late Period		Total	
		n	Pr	n	Pr	N	Pr	N	Pr
Pots	No lugs preserved	26	22	5	13	1	4	32	18
	Two lugs*	4	27	10	16	19	22	33	20
	Four lugs	-		-		22	23	22	23
Total		30	22	15	15	42	21	87	20

*In cases where the pot may have had two or four lugs, it has been counted as having two lugs.

Employing the Acidified Methanol extraction yielded superior recovery rates for both the Early (27%) and Middle Period (32%). These figures stand in contrast to the marginally lower yields obtained from the solvent procedure, documenting rates of 18% and 19% for the respective phases. For the Late Period, all positive samples were obtained from chloroform/methanol extracts. Despite the discrepancy in extraction techniques, these did not account for the observed variations in preservation rates amongst differing pottery forms. Moreover, no substantial difference in lipid concentration between Acidified Methanol extracts and Solvent extracts was detected. This observation suggests comparability between the two extraction methodologies when applied specifically to the case of Barcın Höyük.

**Fig 7:**
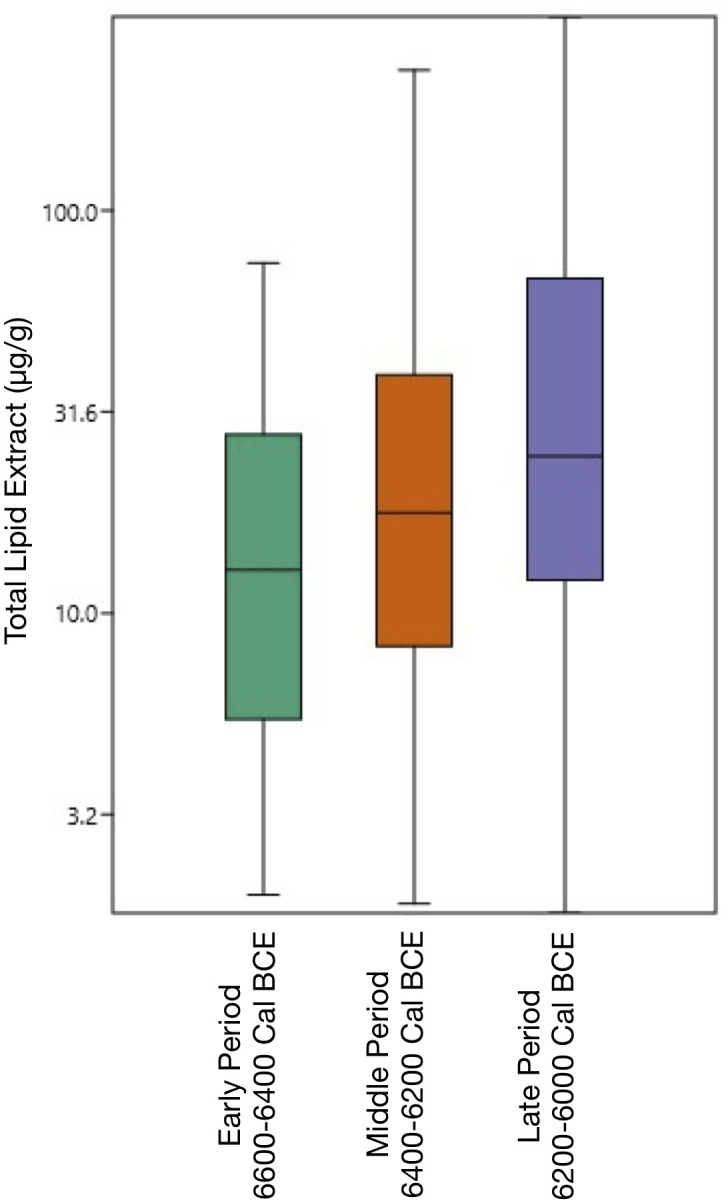
Boxplot presenting the distribution of lipid concentrations per gram of pottery vessel for each analytical Period at Barcın Höyük.

When lipids were detected, their concentrations varied between 1.8 and 303 micrograms of fat per gram of pottery (μg/g). The median total lipid extracts progressively increased throughout each Period: 12.8 μg/g for the Early Period, 17.7 μg/g for the Middle Period, and a peak of 24.55 μg/g for the Late Period. This reveals a consistent rise in recovered fats (Fig 7, S1 File) corresponding with slight increments in preservation rates, as illustrated in Table 2. This upward trajectory in fat concentration persistently echoes across different pot forms. Collared necked pots, for instance, reveal an increase from a median of 13.7 μg/g in the Middle Period to 33.3 μg/g in the Late Period. Similarly, the lipid concentration in oval bowls increased systematically– 15.3 μg/g for the Early Period, 20.5 μg/g in the Middle Period, culminating at 42.2 μg/g in the Late Period, which denotes the peak median lipid concentration in this study. Thus, not only more vessels had lipids, but the quantity of lipids contained also increased through time.

While this constant increase in concentration is statistically significant (Kruskal-Wallis test, H 11.07, p(same) 0.003952)(see S1 File for additional details), it is hard to pinpoint a causal relationship for such trend due to the multiplicity of factors affecting lipid concentration in the pottery such as the intensity of use, the post-depositional conditions and the pottery fabric.

### Biomolecular characterization of the lipid extracts

**Fig 8:**
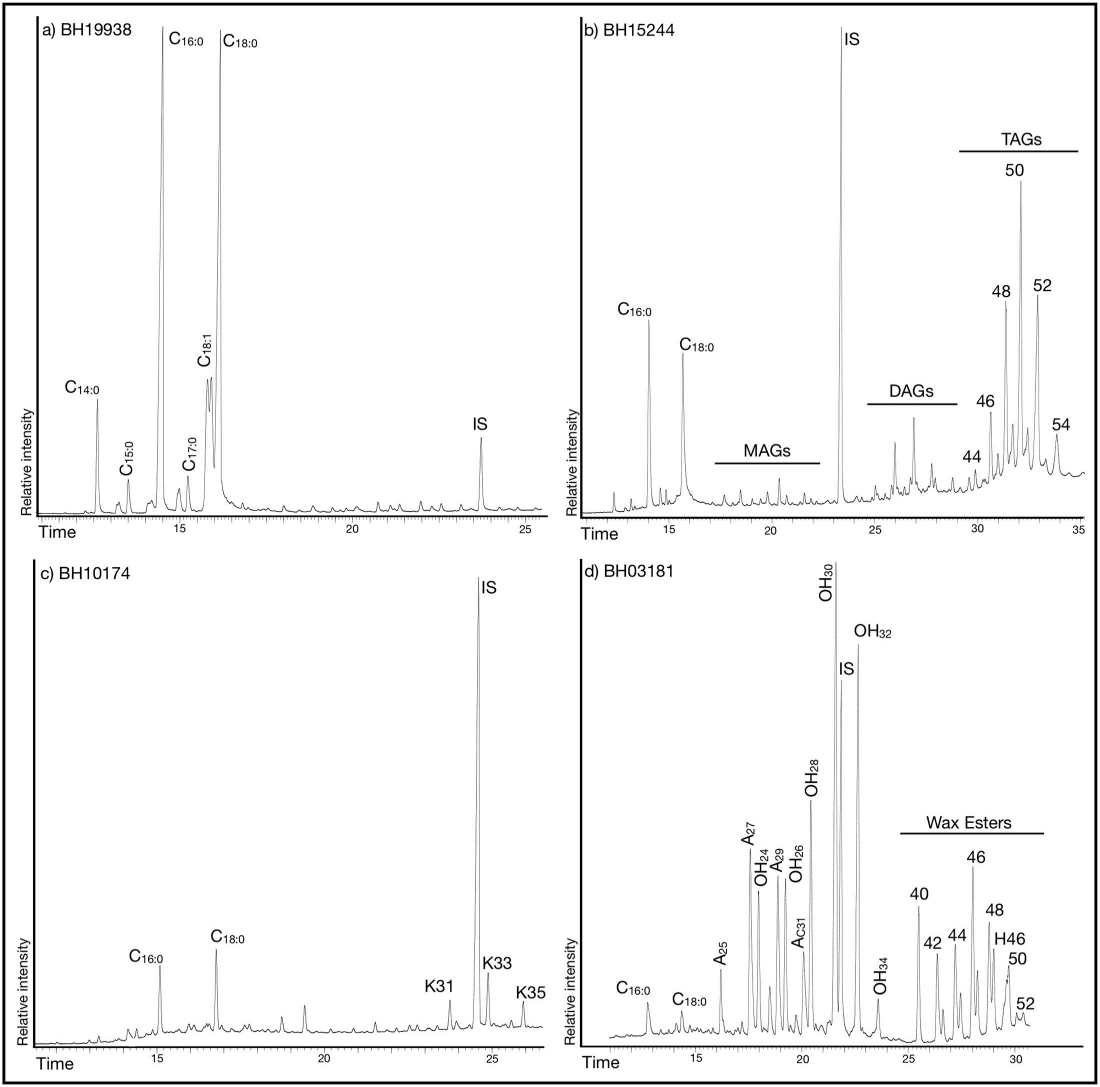
Selected chromatograms from samples in the study as examples for a) free fatty acids (C_x:x_), b) partially hydrolyzed dairy triacylglycerols, c) long chained ketones (K31, K33, K35) demonstrating the protracted heating of palmitic and stearic acids and d) partially hydrolyzed wax including (A_x_) alkanes and (OH_xx_) fatty alcohols, wax esters and hydroxy-wax esters (H_xx_) IS: Internal Standard, *n*-tetratriacontane.

Identified lipid extracts typically comprised a mixture of triacylglycerols (TAGs), diacylglycerols (DAGs), monoacylglycerols (MAGs), and free fatty acids (FFAs) (Fig 8b, S1 Table). The relative intensity of these compounds varied in accordance with each sample’s preservation level. Full TAG profiles, accompanied by partially degraded DAGs and MAGs, were observed in 61 instances, equivalent to 36% of the positively identified results. However, in a majority of samples extracted through the solvent process, as well as those from all acidified methanol extractions, the detected residues predominantly consisted of free fatty acids. Among these, palmitic and stearic acids emerged as the most abundant moieties (Fig 8a), while other compounds such as tetradecanoic, pentadecanoic, heptadecanoic and octadecenoic acids were detected in minor amounts. The palmitic to stearic acid ratio (PS ratio) ranged from 0.3 to 2.7 (median: 0.9, mean: 1.0, standard deviation: 0.43), suggesting that the recovered free fatty acids most probably originated from degraded animal triacylglycerols. Only in one case (BH13410, Phase VId2, Middle Period) a PS ratio of 4 suggested the presence of a plant residue [45,46]. GC-MS analyses additionally detected minor amounts of monounsaturated fatty acids, very long chained fatty acids, dicarboxylic acids and branched fatty acids, common in extensively degraded animal fats and coherent with previously reported animal lipid residues from the Aegean and the Anatolian Neolithic [10–12,47]. Long mid-chained ketones (LCK) indicative of the protracted heating of palmitic and stearic acids [48–50] were detected in four samples (Fig 8c) and, in eight cases, samples presenting odd-chained alkanes and fatty alcohols were accompanied by a series wax esters and hydroxy-wax esters (Fig 8d). No aquatic biomarkers such as ω-(o-alkylphenyl)alkanoic acids 18 to 22 carbons long, or specific isoprenoic fatty acids (4,8,12-trimethyltridecanoic acid) [51,52] were present in the Barcın Höyük extracts, underlining the terrestrial component of the processed foodstuffs in the studied vessels.

Triacylglycerol (TAGs) distributions can serve as a tool to aid in the detection of distinct types of animal fats [53–56]. Fresh ruminant adipose fats present a TAG distribution between C44 to C54 carbon numbers while fresh non-ruminant animals present a shorter range limited between C46 and C54. Furthermore, dairy fats show the widest TAG distributions reaching C40 to C54 carbon numbers in experimental studies where milk was absorbed in unglazed pottery vessels and degraded under oxic conditions [57]. However, the preferential loss of lower molecular weight TAGs such as C40, C42 and C44 due to degradation may yield the dairy TAGs indistinguishable from adipose fats [57] when only the C46 or higher TAGs are preserved. Therefore, while the preservation of the C40 to C44 TAGs could provide complementary evidence for the origin of animal fats, TAGs must be used in conjunction with other criteria such as the compound-specific isotopic analyses of palmitic and stearic acid.

**Fig 9:**
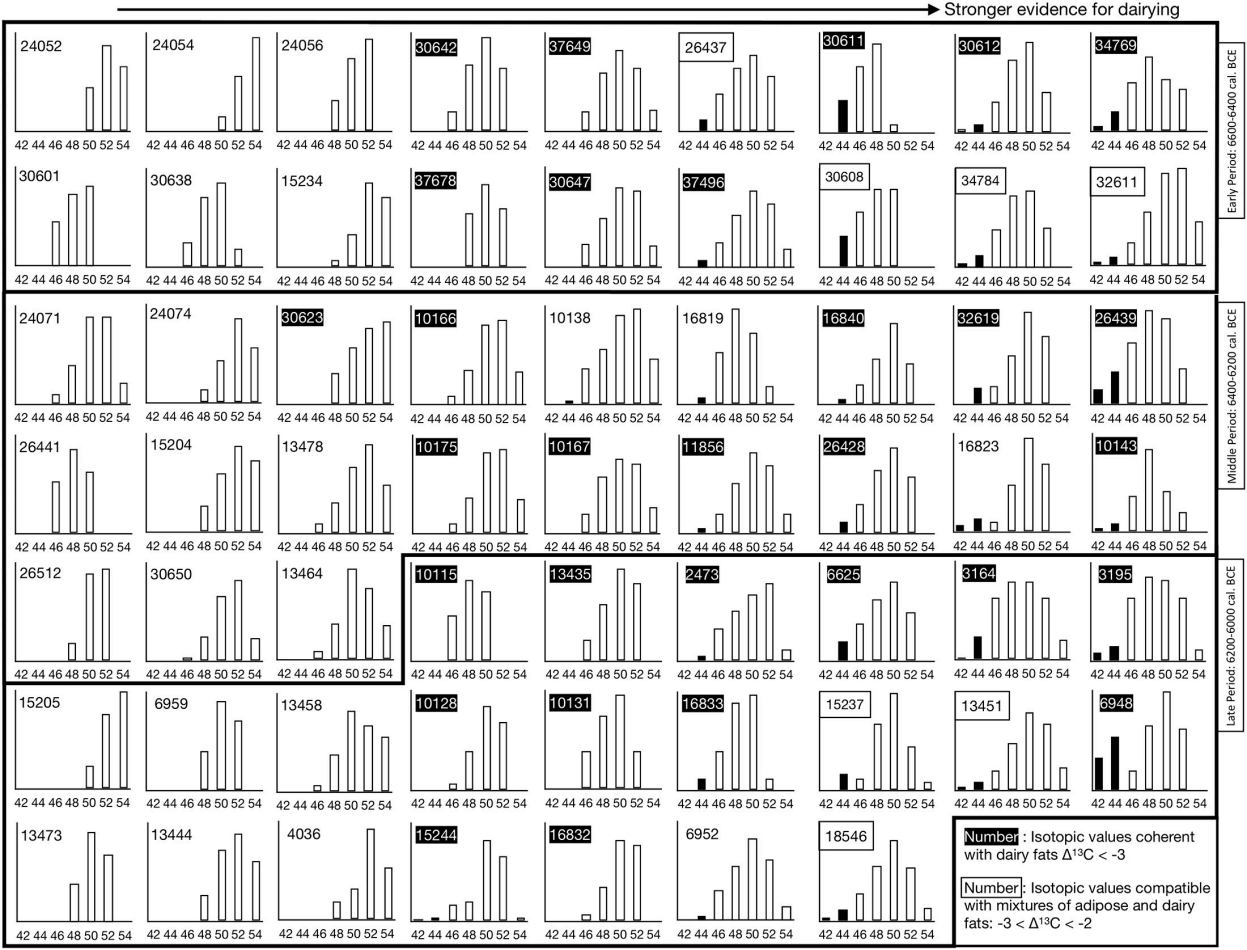
Bar graphs presenting the TAG profiles detected from 61 lipid residues in pottery from the Early, Middle and Late Periods. The Y axis represents the normalized relative abundances of the range of TAGs in each vessel. TAG C42 and C44 bars, potentially deriving from dairy products, are shown in black. Isotopic values for the potential mixing of dairy products with other fats are based on published lineal and bayesian mixing models [58,59]. The abundance of each TAG was calculated by integrating the peaks obtained in the HT-GC chromatograms.

The recovered TAG profiles from Barcın Höyük (Fig 9, S1 File) yielded compounds with 42 to 54 carbon numbers. While in some cases a full range was detected (ex: BH32611, C42 to C54), others presented a minimum of only three triacylglyerols (ex: BH24054, C50 to C54). Across all Neolithic phases at Barcın Höyük, 28 of the 61 vessels (45%) with preserved TAG profiles contained either C42 or C44 moieties, most probably deriving from dairy fats. This represented 44% of the TAG profiles from the Early Period, 42% of those from the Middle Period, and 50% of those from the Late Period, thus suggesting an increase in dairy production and consumption during the final phases of the Neolithic. Given the possibility that highly degraded dairy TAG profiles can be indistinguishable from adipose fats, these percentages were treated with caution and validated with isotopic data.

Furthermore, the presence of triacylglycerols, which is indicative of a high degree of preservation, has only been registered in 8% of all the 805 studied pottery sherds. In the Early and Late Periods, these are coherently distributed across different pottery types according to the frequency in which they were sampled. However, in the Middle Period, 61% of the recovered TAG profiles originated only from bowls (oval and s-shaped) despite these representing only 20% of the studied assemblage for that period. This result is significant. Because bowls yield above average lipid concentrations and high lipid preservation rates we can conclude that this vessel type may have played a special role in the management, transformation and/or consumption of fat-rich products in the VIc, VId3 and VId2 phases (Middle Period) at the site (Table 2).

### Isotopic characterization of the lipid extracts

**Fig 10:**
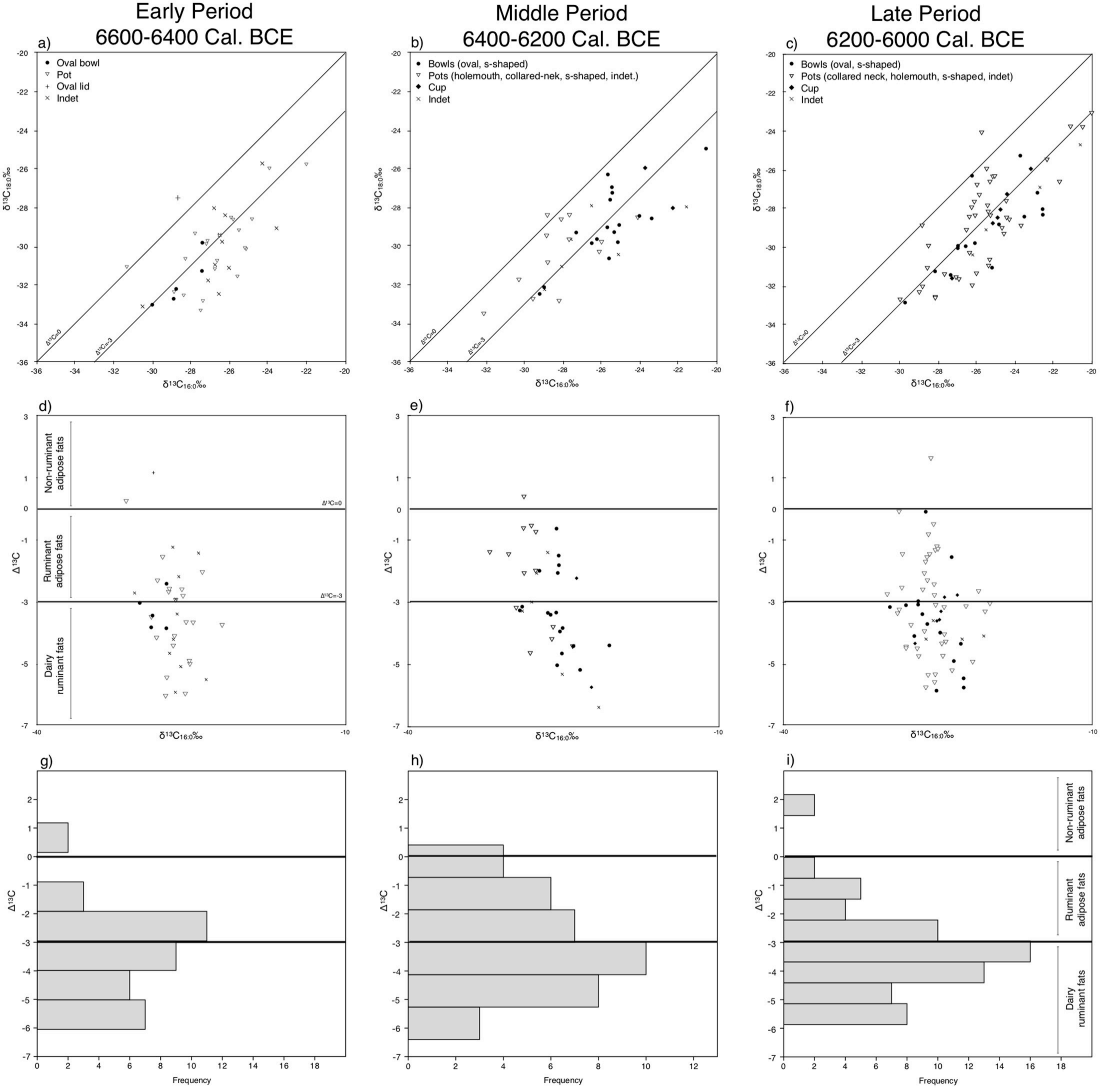
a-c, x/y plot presenting the δ^13^C_C16:0_‰ and δ^13^C_C18:0_‰ values for each vessel, grouped by type and Period. d-f: x/y plots presenting Δ^13^C values and δ^13^C_C16:0_‰ ratios over time. g-i: Histogram of the frequency of Δ^13^C values over time.

Compound specific isotopic analyses of 143 sherds (see the isotopic dataset in S1 Table) showed that, overall, palmitic acid (δ^13^C_C16:0_‰) presented values between -32.1‰ and -20.0‰ with a median of -26.1‰ and a standard deviation of 2.3‰, which is coherent with the range of values detected in other sites from the eastern Mediterranean and the Anatolian Peninsula [10,24,47]. Stearic acid isotopic ratios (δ^13^C_C18:0_‰) tended to be more negative and were placed between -33.5‰ and -23.1‰ with a median of -29.3‰ and a standard deviation of 2.3‰. In consequence, Δ^13^C values (δ^13^C_C18:0_‰- δ^13^C_C16:0_‰), detecting physiological differences in fatty acid biosynthesis between tissues [60] and used to discriminate between non-ruminant adipose (> 0‰), ruminant adipose (< 0‰ > -3.1‰) and ruminant dairy (< -3.1‰) products, were placed between -6.4‰ and 1.7‰ with a median of -3.3‰ and a standard deviation of 1.6 (Fig 10).

The range of isotopic ratios observed across different Periods (Table 4) presented only minor variations. Although median δ^13^C_C16:0_‰ and δ^13^C_C18:0_‰ values were somewhat stable, these tended to become less negative overtime. This trend also affected their respective minimum and maximum values, revealing a generalized shift of at least 1‰ in all the studied summary statistics. Alternatively, Δ^13^C values remained unchanged, with medians lower than -3.3‰, suggesting that the main products contained in the vases were dairy products (Fig 10).

**Table 4:** Summary statistics of the isotopic ratios and proxies obtained in the compound-specific isotopic analyses (minimum, maximum, median and standard deviation).

	Minimum	Maximum	Median	Standard Deviation
**Early Period, 6600–6400 Cal. BCE**				
**δ** ^**13**^ **C**_**C16:0**_ **‰**	-31.3	-22.0	-26.7	1.9
**δ** ^**13**^ **C**_**C18:0**_ **‰**	-33.3	-25.7	-30.1	2.0
**Δ** ^**13**^ **C**	-6.1	1.2	-3.5	1.6
**Middle Period, 6400–6200 Cal. BCE**				
**δ** ^**13**^ **C**_**C16:0**_ **‰**	-31.1	-20.5	-26.2	2.5
**δ** ^**13**^ **C**_**C18:0**_ **‰**	-33.5	-24.9	-29.4	1.9
**Δ** ^**13**^ **C**	-6.4	0.4	-3.2	1.6
**Late Period, 6200–6000 Cal. BCE**				
**δ** ^**13**^ **C**_**C16:0**_ **‰**	-30.0	-20.0	-25.5	2.2
**δ** ^**13**^ **C**_**C18:0**_ **‰**	-32.9	-23.1	-28.6	2.4
**Δ** ^**13**^ **C**	-5.9	1.6	-3.3	1.5

These results imply that, out of 143, 85 vessels presented values coherent with the presence dairy products (60%), 51 sherds may have contained ruminant adipose fats (36%) and only 5 samples yielded isotopic values detected in non-ruminant adipose fats (4%) (Table 5). Across the three periods, dairy products were always dominant. Notably, the Late Period shows a further increase of around 10% in the frequency of milk residues compared with the previous period, which is coherent with the similar rise detected by the TAG profiles. Regarding adipose fats, those originating from ruminant animals where the second most abundant product at Barcın Höyük, although comparisons with TAG profiles suggests some of them might be the result of mixtures with dairy products (BH26437, BH30608, BH32611, BH34784, BH13451, BH15237, BH18546, Fig 8) or mixtures of dairy with non-ruminant products or plant fats, interestingly only detected in bowls from the Middle Period (BH10138, BH16823). The seven instances of potential mixtures of dairy products and ruminant adipose fats appear almost exclusively in closed shapes, namely pots in the Early Period, and two s-shaped pots and one cup (open shape) in the Late Period (See S1.2 in S1 File for more details).

**Table 5:** Frequency of dairy, ruminant and non-ruminant fats in each of the three studied Periods at Barcın Höyük.

	Dairy	Ruminant	Non-Ruminant	Total
**Early Period, 6600–6400 Cal. BCE**	22 (58%)	14 (37%)	2 (5%)	38
**Middle Period, 6400–6200 Cal. BCE**	21 (55%)	16 (42%)	1 (3%)	38
**Late Period, 6200–6000 Cal. BCE**	43 (64%)	21 (31%)	3 (4%)	67
**Total**	85 (60%)	51 (36%)	7 (4%)	143

When considering the various pottery types present in the Early Period (see S1 Table for detail on pottery types and associated results), it became apparent that dairy products are primarily found in pots, specifically holemouths (6, 27%) and variants (6, 27%). Interestingly, oval bowls also notably contained dairy products (4, 18%). In the Middle Period, open forms such as oval (9, 45%), S-shaped bowls (15%), and, for the first time, a cup (1, 5%) became more prevalent in containing the majority of the dairy products. This Middle Period increase in open vessels coincides with a significant decrease in the use of closed vessels (5, 25%) for dairy products. However, in the Late Period, there was a reversal of this trend, with closed forms, specifically pots (13, 30%), dominating as the primary type for dairy products. Nonetheless, milk residues persistently continue to appear in oval (3, 7%) and S-shaped bowls (9, 21%) as well as in cups (4, 9%), suggesting a long-lasting connection with dairy products.

## Discussion

The data from Barcın Höyük demonstrate an intensive use of pottery vessels for cooking and serving foodstuffs that contain adipose fats and dairy lipids as well as (at least in one instance) a potential plant residue. The range of recovered fats is coherent with the different species of animals documented at Barcın Höyük. From the earliest levels onwards, the animal bone assemblage from the Neolithic sequence is dominated by domesticates (more than 90% of the remains). The percentage of cattle remains is similar to ovicaprines but the latter is slightly higher, when the number of identified specimens are considered. Among ovicaprines, sheep was more prolific than goat. Wild or domestic pigs were quite rare in the Neolithic phases. This coincides with the apparent absence of non-ruminant adipose fats in the pottery vessels.

The culling profile for sheep suggests a mixed meat and milk model. Based on postcranial elements, a significant proportion of animals were slaughtered before reaching six months of age, while epiphyseal closure indicates that most ovicaprines survived at least a year. Between 12 and 18 months, the survival rate appears balanced. However, only a few sheep and goats survived beyond four years, with some slaughtered as early as 30 to 48 months. The selective culling of newborn and very young lambs and kids may suggest the exploitation of dairy products, while the overall postcranial material supports the exploitation of meat [4–6].

For cattle, the postcranial culling profiles reveal the slaughter of calves up to six months old. Beyond this age, a majority were retained and survived up to a year, with a significant number surviving up to 20 months. The ratios of fused and unfused specimens, indicative of individuals slaughtered or surviving up to three or four years, appear relatively balanced. Arguing for dairying is notoriously difficult for cattle, but the available evidence does not contradict the possibility of milk being obtained alongside an exploitation for meat, as it has been suggested for other Neolithic societies [61].

Overall, the integration of the faunal data with the recovered lipid signals shows that the high frequency of dairy-based meals cooked with pottery was not achieved through specialized herd management aimed at maximizing milk production. Instead, it is plausible to consider that some of the meat might have been cooked using alternative techniques such as direct roasting over the fire. Most likely, pottery was primarily used for processing milk, indicating a specialized role in the transformation of animal products. However, it is improbable that all pottery, regardless of shape or size, served this purpose, making a careful evaluation of the lipids present in each vessel type necessary.

Upon examining the data, several trends become apparent over time. Despite the limited use of ceramics at the start of the Early Period and an occasional reliance on indirect hot-stone cooking methods [33], even the earliest vessels at Barcın VIe attest the use of high amounts of dairy products. As time progressed, two particular vessel types selected primarily for the storage or processing of dairy became notable: bowls and suspendable four-lugged pots.

Lipid evidence shows that bowls, comprising several different types including oval, hemispherical, collared and S-shaped forms, appear to have been intensively used to either contain, transform, or consume dairy products. In fact, in each of the three Periods, 70–80% of all bowls presenting lipids would have been consistently reserved for this purpose. Furthermore, some of the bowls recovered yielded evidence for interior and exterior smudging. At least one of the open bowls from the Late Period (BH13443), yielding an isotopic signature indicative of milk, had evidence for long chained ketones near its rim, a heating signature confirming that the milk fats in bowls may have been heated. While these vessels could have been used for a wide range of everyday cooking activities, it is also highly probable that they were involved in the preparation and integration of milk/dairy into daily meals. In other words, some processing of high-lactose raw-milk would have been crucial for the inhabitants of Barcın Höyük, who would have been mostly lactose intolerant [62] but nonetheless reliant on a significant amount of milk/dairy in their everyday lives. It is interesting to note that yoghurt making requires the heating of milk to 85-90ºC before an incubation period of 8 hours at around 40ºC, a practice still traditionally performed in the region around Barcın Höyük today using precisely this type of open straight-sided vessels [63].

**Fig 11:**
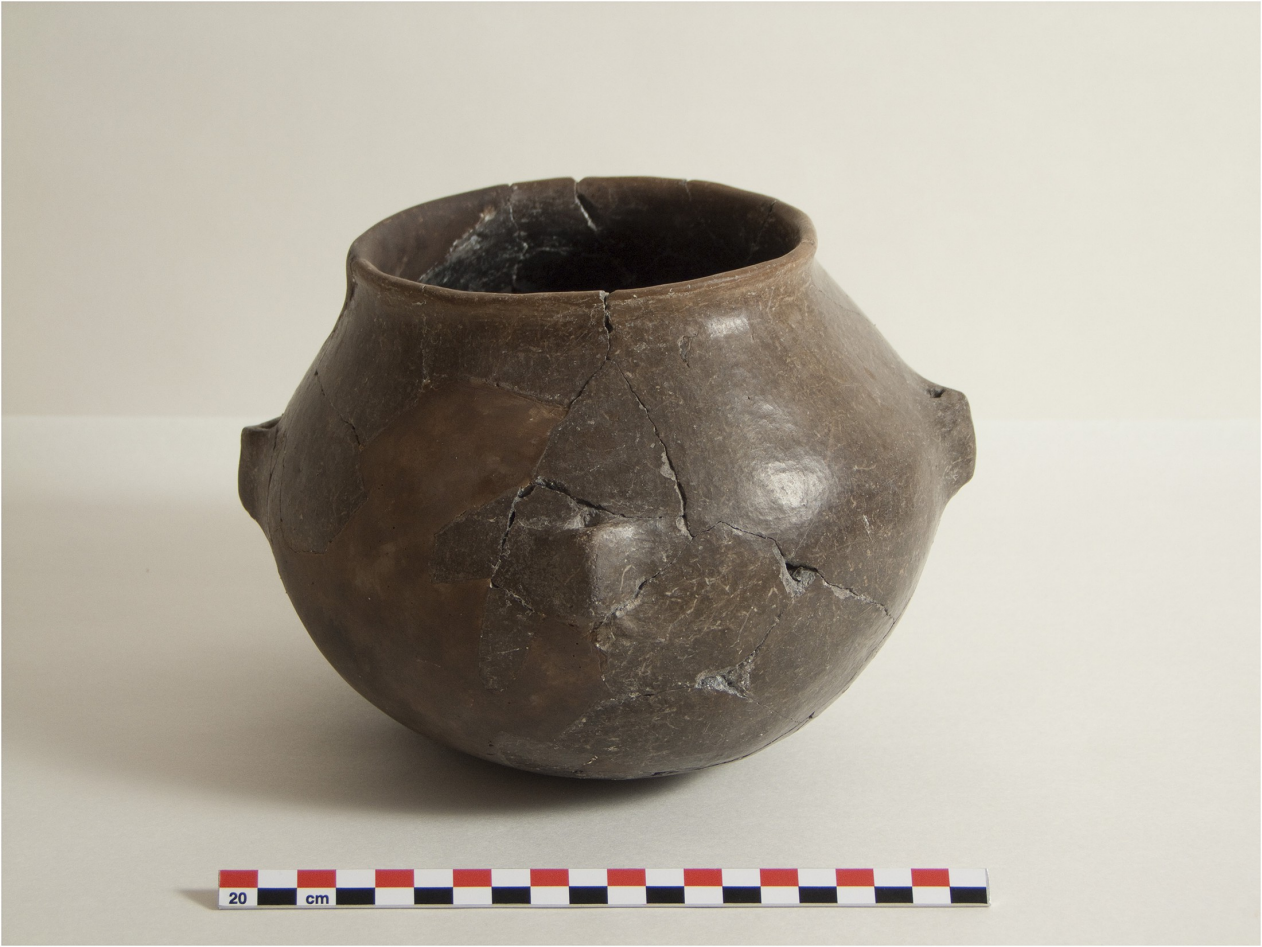
Complete four-lugged Late Period pot from Barcın Höyük.

The quantity of oval bowls in the Early Period is relatively low and only seven of them yielded identifiable residues in VId1, but the persistence of this category through time and the consistent evidence for dairy residues ranging around 70–80% confirms its importance for the processing of dairy products. In essence then, given these data, we may be able to argue that the inhabitants of Barcın Höyük used bowls for dairy products from the earliest levels onwards. This may potentially have taken place in conjunction with heating closed shapes like pots, which were undeniably a category associated with dairy products, albeit in a different way than bowls. In the Early Period, the proportion of ruminant adipose fats and dairy products were similar in pots, including primarily holemouth shapes, suggesting that meals involving both ingredients may have been prepared in this vessel type. By the Middle Period, there is a noticeable increase in the selection of two-lugged pots to preferentially contain ruminant adipose fats, relegating dairy residues to a second position. Nonetheless, the Late Period presented an innovation, the appearance of four-lugged pots with vertically-pierced lugs, facilitating the stable hanging of the vessel (Fig 11). While nine of the 18 (50%) holemouth and two-lugged pots in the Late Period contained milk residues, when it comes to the newly introduced four-lugged pots, 16 of the 23 (69.6%) yielded either isotopic or triacylglycerol evidence of dairy products. A One-sample Non-parametric Chi-Square test for independence suggests that the distribution for the Late Period comparing four-lugged pots to the distribution of two and non-lugged pots is statistically significant: χ2 (2, N = 23) = 7.913, p = 0.019) showing that these vessels were produced and intended for a purpose that differs from other pots. In four-lugged pots, when the total amount of fat recovered is taken into account, 741 μg/g of lipids originated from dairy products and only 230 μg/g were attributed to adipose fats (182.7 μg/g from ruminants and 47.1 μg/g from non-ruminants). This represents 76% of all the recovered lipids for this pottery type and suggests that four-lugged pots were presumably more in contact with milk-based fatty rich substances, thus generating dairy residues with higher concentrations of lipids.

**Fig 12:**
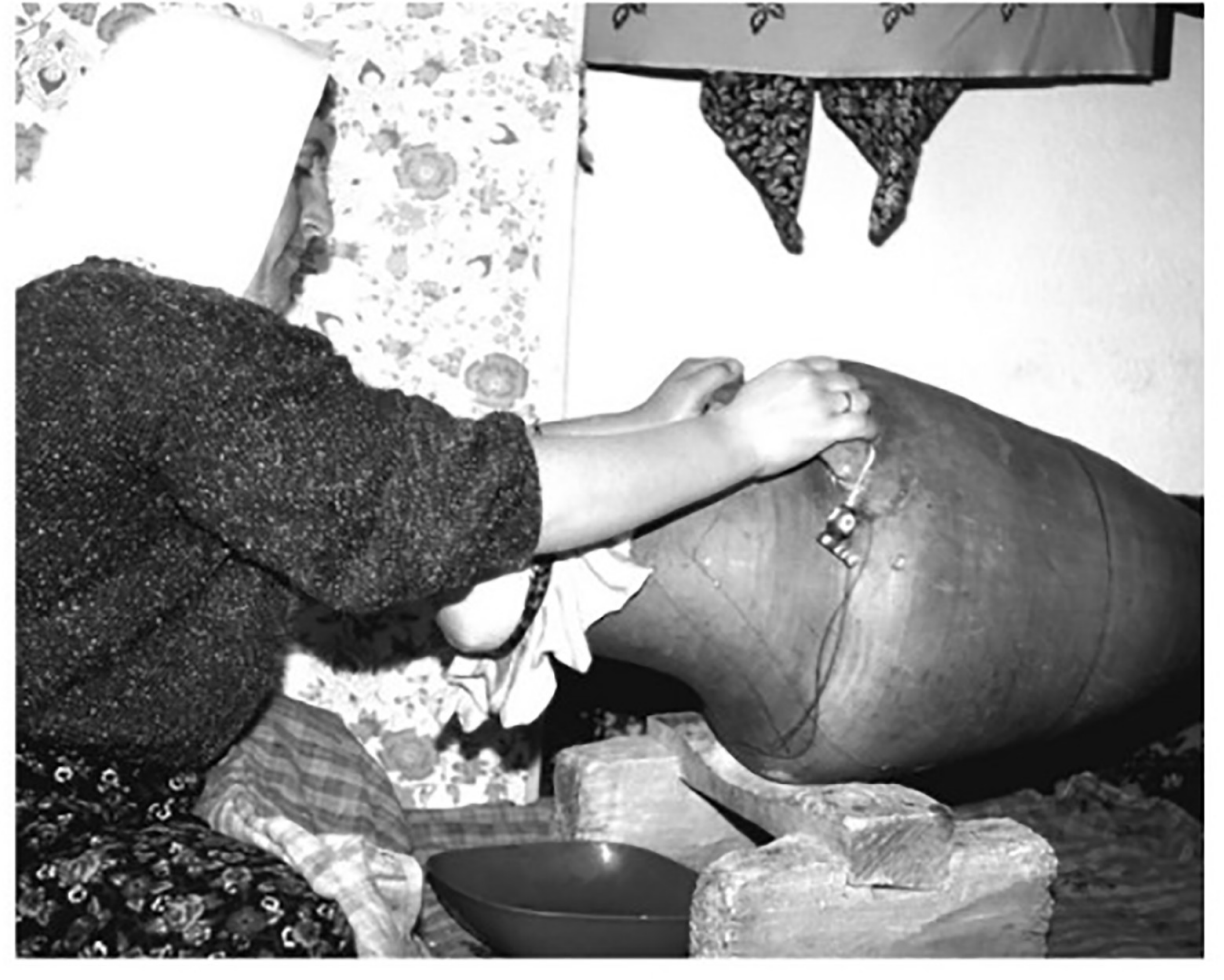
Ethnographic case of using a ceramic churn (Kızılkaya, early 1990s). Image reproduced with permission from Füsun Ertuğ [68].

Although two-lugged vessels with pierced handles were already present in the Middle Period, the invention of four-lugged pierced vessels would have enabled stabilization of the pot when hanging, preventing the contents from spilling. Such pots with a suspension mechanism would have been ideal for churning milk. Churning thickens milk through physical agitation, which in turn creates fissures in the milk fat membranes. These fat globules can stick to each other and clump up to create thickened products like cream, butter and butter milk or *ayran*-type drinks [64]. There is a significant corpus of ethnographic and archaeological research suggesting that churns made of pottery or wood, some of which has suspension mechanisms, were used across Anatolia through time [65–68] (Fig 12). In Barcın Höyük, four-lugged vessels could have been used above a fire but they may have also been secure enough for swinging as part of the churning process, thus becoming excellent shapes to process dairy products.

The occupation at Barcın Höyük ended at around 6000 BCE, but pots with four suspension lugs became an indispensable part of the late seventh and early sixth millennium ceramic assemblages from the Marmara Region, being found in sites such as Fikirtepe and Pendik [69], Ilıpınar [70] and Yenikapı [71], and also further afield among Balkan Early Neolithic sites [72]. Overall, the four-lugged pot that emerged as a specialized dairy vessel type around 6200 cal BCE at Barcın Höyük may have become the typical dairy processing vessel during the Early Chalcolithic in the Marmara Region. Then, dairying traditions and associated material cultural aspects that had their roots in the Marmara Region may have spread westwards following the Neolithization process. Future lipid studies on four-lugged pots within northwestern Anatolia and southeastern Europe will thus be necessary to better understand the role this vessel type had in the area. Current publications from these regions present similar (Marmara) or lower (Greece and the Balkans) percentages of dairy products than Barcın [10,24,73], but a direct comparison is impossible as lipid data is not linked to pottery types.

## Conclusion

The study of 805 vessel fragments from Barcın Höyük has shown that the production, transformation, and consumption of dairy products was already prevalent in Northwest Anatolia by 6600 cal. BCE, at the end of the first half of the seventh millennium cal. BCE (Early Period), constituting one of the oldest pieces of evidence on dairying in Anatolia and North Mesopotamia to date. Assuming that milking ruminants would have been a seasonal activity—and considering the absence of the lactase persistence gene amongst Neolithic populations—the extensive consumption of dairy products detected at Barcın Höyük is best explained by the involvement of pottery vessels of specific shapes in the practice of milk-transformation strategies, such as the preparation of dairy products.

Dairying should be acknowledged as a practice where farm animals contribute to various benefits beyond just providing meat. The fully developed dairy economy documented in the oldest layers at Barcın Höyük suggests, however, that the earliest inhabitants of the site must have had some knowledge about dairying prior to their arrival in the Yenişehir Valley. The only other Anatolian site to date reporting contemporaneous evidence for cooking with dairy products during the first half of the seventh millennium is Çatalhöyük [10,24,74]. Within this time frame, the abundance of dairy products at Çatalhöyük in the form of lipid residues is significantly lower than those at Barcın Höyük, suggesting that an incipient dairying stage of experimentation may have occurred east of the Marmara Region. Nonetheless, additional data will be required to assess the relevance of dairy products for the inhabitants of the core Neolithic regions, where milk may have played a role in providing the necessary food security to foster the expansion of the Neolithic way of life into unknown landscapes further west [75].

At Barcın Höyük, starting in early times onwards, much of the processing was performed in open-shaped vessels such as oval and S-shaped bowls while pots may have been primarily used in everyday cooking, where dairy products were mixed with adipose fats. The intensity of dairy production was stable across the second half of the seventh millennium at Barcın Höyük (the Early and Middle Periods), although some later innovations, including the four-lugged pot, may have fostered an increase in the availability and variability of dairy-based recipes after 6200 cal. BCE (the Late Period). Precisely, the widespread presence of four-lugged pots across the Marmara Region at the end of the seventh and the beginning of the sixth millennium is indicative of the successful expansion of a toolset specialized on dairy production, which may have aided in the success of dairying in the region and thus the first widespread adoption of dairy products by humans.

## Supporting information

S1 File Supplementary information. (DOCX)

S1 Table Dataset of vessel characteristics and biomolecular and isotopic results. (DOCX)
