# Supplementary material for: From bowls to pots: The dairying revolution in Northwest Turkey, a view from Barcın Höyük, 6600 to 6000 BCE
Source: PLoS One. 2024 May 9;19(5):e0302788. doi: 10.1371/journal.pone.0302788 (PMC11081328; doi:10.1371/journal.pone.0302788)
Supplement: S1 File — (DOCX) [file pone.0302788.s001.docx]

**S1 Supplementary information**

**1.1 Additional details on the sampling strategy**

Regarding vessel type, PHASE 1 had the lowest percentage of diagnostic sherds given the scarcity of ceramics in the earliest levels. Nonetheless, 178 samples (68%) yielded identifiable profiles enabling form designations. In this level, closed forms were exclusively pots (N= 135), including holemouth pots (N= 67) collared neck pots (N= 2) and two-lugged pots (N= 15). The most prevalent open forms were oval (N= 18) bowls but collared (N= 3) and hemispherical (N= 6) bowls as well as dishes (N= 3), lids (N= 8), boxes (N= 3) and a cup were also present in the studied assemblage (Tables 1 and 2 ).

In PHASE 2, vessel type was determined for 174 samples (75%) these included closed forms (N= 100, 57%) most of which presented two lugs (N= 61, 61%) such as collared-neck pots (N= 50), holemouth pots (N= 17) and S-shaped pots (N= 4). Open forms (N= 68, 39%) included cups (N= 6) and a dish (N= 1) and were dominated by bowls of several types: S-shaped (N= 24), collared (N= 7), hemispheric (N= 6) and oval (N= 24). Other categories included boxes (N= 2) and lids (N= 4).

In PHASE 3, vessel type was available for 272 samples (87%); only a small percentage of the sherds sampled were non diagnostic or not attributable by form. In this phase, the assemblage was again dominated by closed types comprising 75% of the (N= 204) vessels. For the first time, this phase shows a significant innovation, the majority of the studied pots presented four lugs (46.5%). This was combined with different profiles including holemouth pots (N=11), collared neck pots (N=52) and
S-shaped pots (N= 44). Open forms (N= 66, 24%) included S-shaped bowls (N= 32), oval bowls (N= 12) and a single hemispheric bowl. This was complemented by one box fragment and one miniature vessel.

| Vessel category/type | | 1  VId1, Vie | | 2  VIc, VId3, VId2 | | 3  VIa VIb | | Neolithic  Total | |
| --- | --- | --- | --- | --- | --- | --- | --- | --- | --- |
|  |  | N | % | N | % | N | % | N | % |
| Closed | Pot (not preserved, two or four lugs) | 65 | 37 | 29 | 16.5 | 97 | 35 | 191 | 32 |
|  | Holemouth pot (none or two lugs) | 67 | 38 | 17 | 9.5 | 11 | 4 | 95 | 15 |
|  | Collared neck pot (none or two lugs) | 2 | 1 | 50 | 28 | 52 | 19 | 104 | 17 |
|  | S-shaped pot (none or two lugs) | 0 | 0 | 4 | 2 | 44 | 16 | 48 | 8 |
| Open | Oval bowl | 18 | 10 | 24 | 14 | 12 | 4 | 54 | 9 |
|  | Hemispheric bowl | 6 | 3 | 6 | 3 | 1 | 0.5 | 13 | 2 |
|  | Collared bowl | 3 | 1.5 | 7 | 4 | 0 | 0 | 10 | 2 |
|  | S-shaped bowl | 0 | 0 | 24 | 14 | 32 | 12 | 56 | 9 |
|  | Dish | 3 | 1.5 | 1 | 0.5 | 0 | 0 | 4 | 1 |
|  | Cup | 1 | 0.5 | 6 | 3.5 | 21 | 8 | 28 | 4 |
| Special forms | Lid | 8 | 4.5 | 4 | 2 | 0 | 0 | 12 | 2 |
|  | Box | 3 | 1.5 | 2 | 1 | 1 | 0.5 | 6 | 1 |
|  | Miniature vessel | 0 | 0 | 0 | 0 | 1 | 0.5 | 1 | 0.2 |
| Category/type Subtotal | | 178 | 100 | 174 | 100 | 272 | 100 | 623 | 100 |
| Indeterminate | | 85 |  | 59 |  | 38 |  | 183 |  |
| Total | | 262 |  | 233 |  | 310 |  | 805 |  |

Table 1: Sampling program for each vessel type and period. N reflects the number of vessels sampled and % presents the percentage of each type calculated from the category/type subtotal in each period.

| Lugs in pots | | 1  VId1, Vie | | 2  VIc, VId3, VId2 | | 3  VIa VIb | | Total | |
| --- | --- | --- | --- | --- | --- | --- | --- | --- | --- |
|  |  | n | % | n | % | n | % | n | % |
| Pots | No preserved lugs | 120 | 89 | 39 | 39 | 21 | 10.5 | 180 | 41 |
|  | Two lugs* | 15 | 11 | 61 | 61 | 88 | 43 | 164 | 37 |
|  | Four lugs | 0 | 0 | 0 | 0 | 95 | 46.5 | 95 | 22 |
| Total | | 135 | 100 | 100 | 100 | 204 | 100 | 439 | 100 |

Table 2: Number of samples associated with pots whose type include none, two or four lugs. *In cases where the pot may have had two or four lugs, it has been counted as having two lugs.

**1.2 Details and statistical tests on the concentration of lipids in pottery across time.**

In this study, the Lipid concentration per gram of pottery was distributed non-parametrically, with significant amounts of pots containing no detectable fats and only less than 30% of the sherds containing a lipid signal.


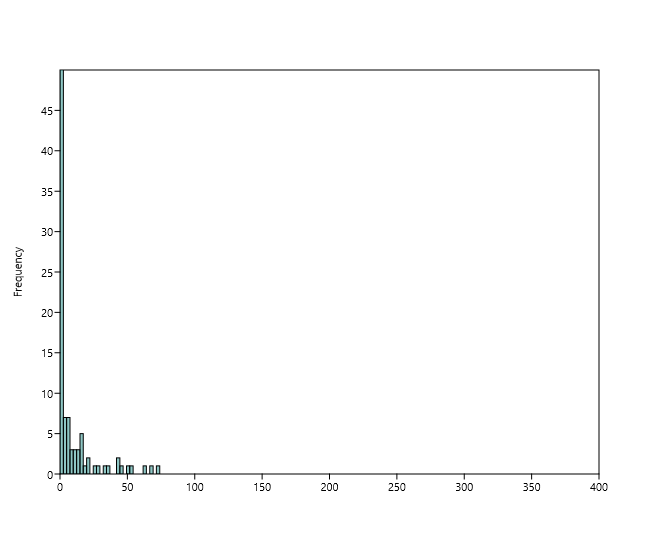

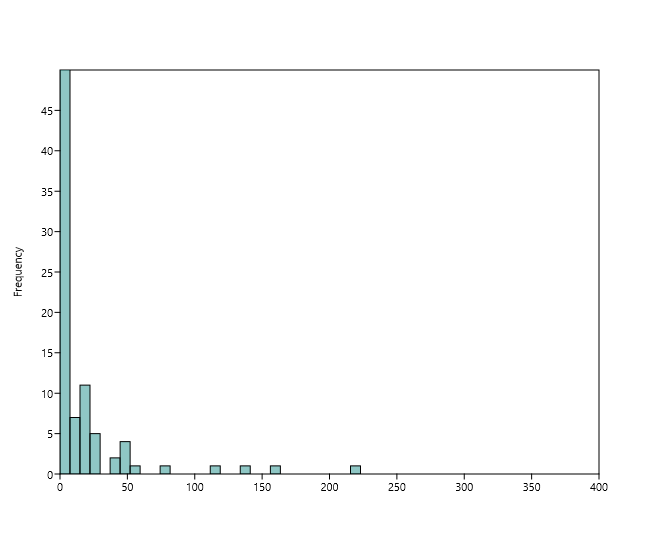

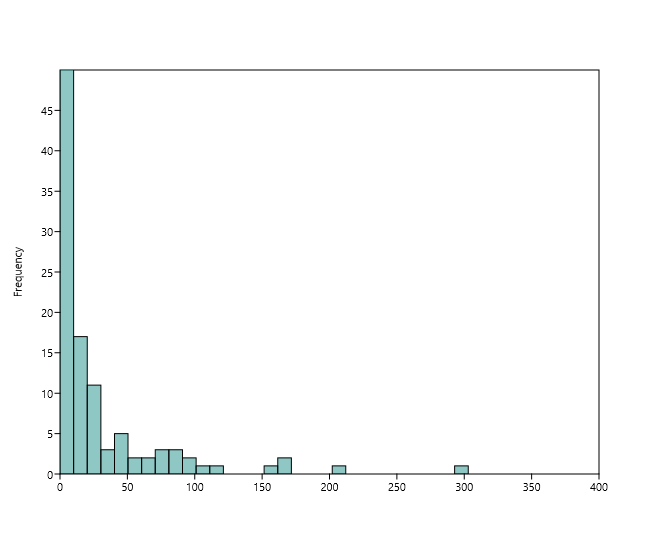


Early phase Middle phase Late phase

Statistical tests to examine the potential increase in lipid concentration over time included a Kruskal-Wallis test for equal medians and its corresponding Mann-Withney pairwise table. Calculations were performed using the PAST4.13 software.

| Test | Statistic | P(same)^1^ |
| --- | --- | --- |
| Kruskal-Wallis test for equal medians | 11.07 | 0.003952 |

1: A significant difference between sample medians is assumed when the p value is lower than 0.05.

Mann-Withney pairwise:

| Mann-Whitney U/  P(same)^1^ | Early | Middle | Late |
| --- | --- | --- | --- |
| Early |  | 856 | 939.5 |
| Middle | 0.1565 |  | 1216 |
| Late | 0.001062 | 0.07455 |  |

The pairwise tests show that the difference between the Early and the Middle periods is statistically non-significant. However, the p values drop for the late phase, where it shows a significant difference with the values from the early period.

**1.3 Additional details on TAG profiles and potential mixtures**

To explore the presence of mixtures of dairy products with ruminant and/or non-ruminant adipose fats, we compared the obtained Δ^13^C values with a set of variables derived from the TAG profiles: the combined presence of the C42 and C44 TAGs; the combined presence of the C42, C44 and C46 TAGS; the TAG average carbon number and the TAG dispersion factor, calculated according to [1].

Presence of TAGS C42 and/or C44 Presence of TAGS C42, C44 and C46


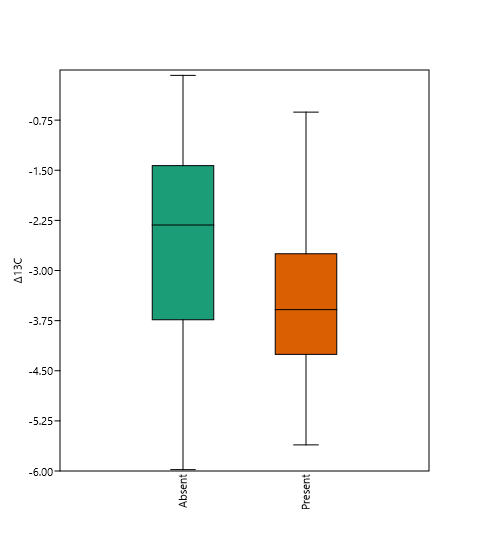

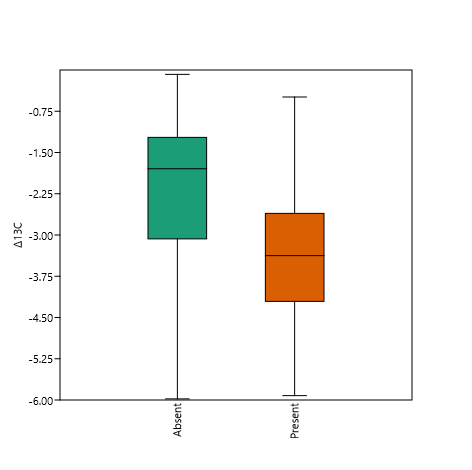


In both cases, the samples containing short-chained TAGs presented a significantly more negative distribution of Δ^13^C values which is coherent with dairy products, as shown in table 3:

|  | Δ^13^C | Descriptive statistics | | Test for equal means | | |
| --- | --- | --- | --- | --- | --- | --- |
|  |  | Mean | Std. Dev. | N | Eq. Var p^1^ | Student T p^2^ |
| TAGs C42 and C44 | Presence | -3.6‰ | 1.2‰ | 58 | 0.13936 | 0.016937 |
|  | Absence | -2.7‰ | 1.6‰ |  |  |  |
| TAGs C42, C44 and C46 | Presence | -3.3‰ | 1.3‰ | 58 | 0.44125 | 0.009957 |
|  | Absence | -2.2‰ | 1.6‰ |  |  |  |

1: Equal variances assumed when the p value is higher than 0.05. 2: Statistically different means are assumed when the p-value is lower than 0.05.

The TAG M and DF values appear to be correlated, showing the presence of either a narrow set of long chained TAGs or a wider set of narrower ones. Furthermore, the average carbon number (M) shows that values lower than 50.1 are, in most cases, coherent with an isotopic signal of dairy products.


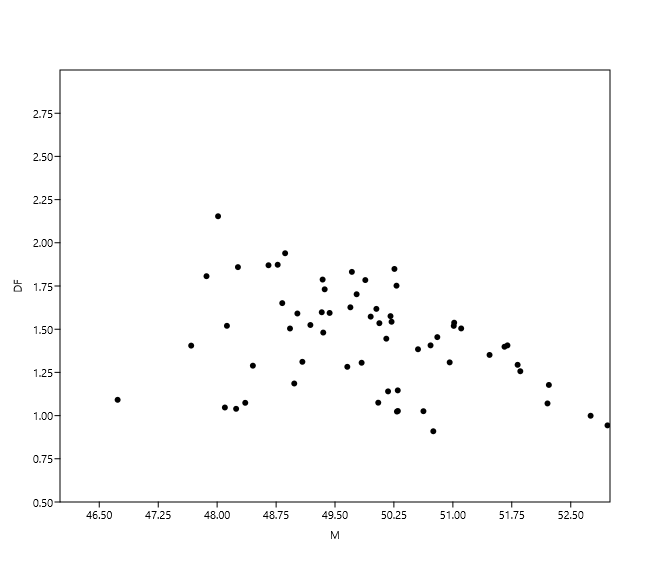

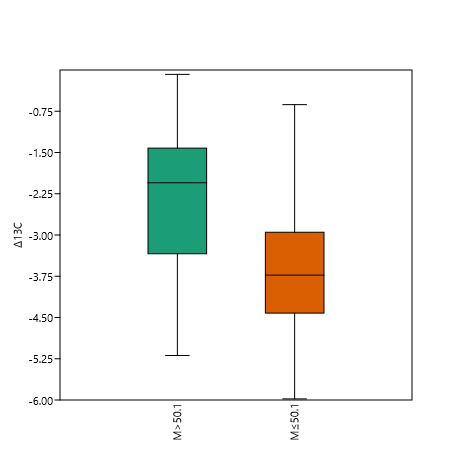


|  | Δ^13^C | Descriptive statistics | | Test for equal means | | |
| --- | --- | --- | --- | --- | --- | --- |
|  |  | Mean | Std. Dev. | N | Eq. Var p^1^ | Student T p^2^ |
| Average Carbon Number (M) | M≤50.1 | -3.7‰ | 1.3‰ | 58 | 0.8043 | 0.00027862 |
|  | M>50.1 | -2.4‰ | 1.3‰ |  |  |  |

1: Equal variances assumed when the p value is higher than 0.05. 2: Statistically different means are assumed when the p-value is lower than 0.05.

In 9 samples, despite Δ^13^C isotopic values above the -3.1‰ threshold, a low M value and the presence of C42 and or C44 TAGs suggests a potential mixture of adipose fats and dairy products. In three samples, BH15237, BH26437 and BH30608, C42 is absent and thus the detected TAG profile could also be coherent with a very-well preserved ruminant fat. In samples BH13451, BH18546, BH32611 and BH34784 the presence of C42 clearly shows that an input of dairy products mixed with ruminant adipose fat is necessary to explain the result. In this cases, mixing models [2,3] have demonstrated that a presence of 30% to 60% of ruminant adipose fat in a mix with dairy products can result in isotopic values between -3.1 to -2, which is the range detected here. Finally, in two cases, BH 10138 and BH16823, with Δ^13^C isotopic values of -1.8‰ and -0.6‰ respectively, an mix of 50% to 60% non-ruminant or plant fats with dairy products might best explain the obtained result.

It is interesting to note that potential mixtures between dairy and ruminant fats are constrained to closed shapes, mainly pots and one cup. However, the two samples presenting a potential mixture with non-ruminant fats or plants were detected in two bowls.

**2 Extractions:**

For this research, two different extraction procedures were selected in order to combine the capability of recovering triacylglycerol profiles and non-hydrolysed lipids of the chloroform/methanol extraction and the higher lipid yields and GC-C-IRMS-ready extracts of the acidified methanol extraction. Although the acidified methanol extraction indeed resulted in a higher percentage of sherds yielding interpretable amounts of lipids, no meaningful differences were detected in the amount of lipids recovered or the isotopic values of one or the other procedure.

**Chloroform/Methanol procedure**

Approximately 2g of the sherd was taken and the surface cleaned using modelling drill to remove any exogenous lipids. The sherd was ground in a pestle and mortar to a fine powder. For solvent extractions, about 2 g of ground ceramic from the inner wall of the sherds were extracted with ca. 10 mL of Chloroform (TCM)/methanol (MeOH) (2:1 v/v) and 20 µg of internal standard *(n*-tetratriacontane) was added. Based on Evershed et al., (1990), after 15 min of sonication and 20 min of centrifugation at 2,500 rpm, the supernatant was removed. This procedure was repeated 2 times, and the 2 extracts were combined. The extract was then dried under a gentle stream of nitrogen, derivatised with 100 μL of N,O-bis(trimethylsilyl)trifluoroacetamide (BSTFA) with 1% trimethylchlorosilane (TMCS) at 70°C for 1 hour and dried. Finally, the sample was redissolved in hexane, transferred to a GC vial.

**Acidified Methanol procedure**

The acidified methanol extractions were performed on 1g of ground pottery prepared in the same way as per the chloroform/methanol procedure, and spiked with internal standard 10 μg of *n*-heptadecane. Following Correa-Ascencio and Evershed (2014). Five mL of an acidified methanol solution (H_2_SO_4_-M_e_OH, 3%, v/v) were added to the sample. The mixture was heated at 70 °C for 1h, then left to cool at room temperature. After this reaction, the pH was checked and, in cases where it was higher than 3, the previous step was repeated. The solution was then separated from the pottery powder by centrifugation at 2500 rpm for 10 min. The clear solution was decanted, and 1 mL double-distilled extracted water was added as the original extracted sample was treated with 2 x 3 mL hexane. Following this, 3 X 2 mL hexane were added directly to clear solution and was mixed in the mixer to extract any remaining residue. Hexane layers were collected to a clean vial and evaporated to dryness under nitrogen gas. So formed FAME derivatives of hydrolysed lipids are injected to GC after dissolving the residue in the vial with 50 µl hexane.

**Boron Trifluoride procedure**

Before preparation of FAMEs, the TLE (Total Lipid Extract) was saponified to release the free fatty acids from acylglycerols in TLEs. For saponification, the TLE was heated with 2 ml 5% (w/v) methanolic sodium hydroxide at 70°C for one hour. Following the neutralisation with 3 M HCL, lipids were extracted into hexane (2x3ml) and the solvent was reduced under a gentle stream of nitrogen at 40ºC. Then, FAMEs were prepared by reaction of free fatty acids with boron trifluoride (BF3) in methanol (14% w/v), at 70ºC for one hour. Then methylester derivatives were extracted with diethyl ether or chloroform and dried under a gentle stream of nitrogen at 40ºC. Before GC injection, the extracts were resuspended in hexane.

**3 Analytical parameters:**

**GC-FID parameters -Boğaziçi University**

**YL 6100C Gas Chromatograph**

BSTFA derivatised TLEs were analysed with an YL 6100C GC instrument equipped with on-column injection port, an FID (Flame Ionisiation Detector) detector and a 15m HT DB-1 column. After injection of 1 to 1.5μl, the oven was kept at 50ºC for 2 minutes and then the temperature increased at 10ºC/min up to 350ºC, where it was held for 20 additional minutes. The FID detector was kept at 350ºC and nitrogen was used as the carrier gas. Standard fatty acids and modern dairy, ruminant and non-ruminant fats were used as references to identify peaks in the GC chromatograms.

**ATI Unicam 610 Series**

Acidified methanol samples were initially analysed with an Unicam 610 Gas Chromatograph equipped with a Flame Ionisation Detector (FID) and a split/splitless injector kept at 250ºC. The analyses were performed on a Carbowax column (30 m length, 320 μm internal diameter, 0.25 μm film thickness) and nitrogen was used as a carrier gas at a column head pressure of 30 psi. After injection of 1 to 1.5μl, the oven was kept at 50ºC for 2 minutes and then the temperature increased at 10ºC/min up to 240ºC, where it was held for 15 additional minutes. The FID detector was kept at 350ºC. Standard fatty acids and modern dairy, ruminant and non-ruminant fats were used as references to identify peaks in the GC chromatograms.

**Agilent 7820A**

Samples were analysed with a 7820A Agilent Gas Chromatographer (GC) fitted with a Flame Ionisation Detector (FID). The injection was done in splitless mode at a temperature of 300ºC and eluted trough an DB-1 capillary column (30 m length, 250 μm internal diameter, 0.25 μm film thickness) using nitrogen as the carrier gas. The oven temperature was initially set at 50ºC for 2 minutes and then increased at 6ºC min^-1^ to 320ºC, where it stayed for 20 minutes. GC-FID analyses were used to study the concentration of fats in the sample and detect the presence and relative abundance of palmitic and stearic acids by comparison with modern reference standards.

**GC-MS parameters – Koç University**

1µl was injected into an Agilent 6890N coupled to an Agilent 5973Network Mass Spectrometer. The GC was fitted with a DB-1 column measuring 30m x 250µm x 0.25µm. The GC injector was operated in splitless mode at 300ºC and helium was used as the carrier gas. The oven temperature was set at 50ºC for 2 minutes and then increased at 10ºC min^-1^ to 300ºC and held at that temperature for 15 minutes. The Mass Spectrometer was run in electron impact mode and masses were acquired in full scan mode between m/z 50 to m/z 600.

**GC-C-IRMS parameters -TÜBITAK (UME and MAM laboratories)**

For samples extracted from the year 2008 to the year 2010, compound specific isotopic analyses were performed in the MAM facilities. For the samples extracted from the year 2010 to the year 2017, compound specific isotopic analyses were performed in the UME facilities.

1µl was injected into a Thermo Finnigan GC Ultra connecter to a Thermo Finnigan MAT 253 mass spectrometer through a Thermo Finnigan GC Combustion IIII interface kept at around 900ºC. Faraday cups were used to detect ions *m/z* = 44, 45 and 46. The splitless injector was kept at around 220ºC and 1µl of sample was injected to Carbowax column (60m x 320 µm x 0.25 µm) and helium was used as a carrier gas. The oven was initially kept at 50ºC for two minutes and then increased 15ºC/min to 150ºC, then at 4ºC/min to 240 and a final isothermal period of 10 minutes. The Instrumental analytical error was stablished at 0.3‰.

In all cases, results have been presented in the standard notation relative to the Vienna Pee Dee Belemnite (V-PDB) standard. δ13C‰ = (R_sample_ − R_standard_) / R_standard_ has been applied for the correction. Samples were additionally corrected for their methylation using a mass balance calculation taking into account the isotopic value of the methanol used for derivatisation.

| **Shape** | | | **N** | **Avg. Dimensions** | | **Lipid amounts** | | **Animal Fats** | | | **Other Biomarkers** | **Shape interpretation** |
| --- | --- | --- | --- | --- | --- | --- | --- | --- | --- | --- | --- | --- |
|  |  |  |  | **Diameter** | **Thickness** | **Preservation rate** | **Total Lipid Extract** | **Dairy** | **Ruminant** | **Non-Ruminant** |  |  |
| Closed | Pot | Holemouth | 95 | 18±2.4 | 8±1.8 | 16% | 45±26 | 6 60% | 2 20% | 2 20% |  | Mid-size type with low preservation rates but high amounts of lipids (in positive cases). Tend to mostly be used to consume dairy but also other fat types. |
|  |  | Collared-neck | 104 | 20±3.8 | 8±2.0 | 11% | 26±35 | 4 44% | 5 56% | 0 0% | Mixtures of dairy and ruminant fats | Big vessels yielding mixtures of fats. Only shape where ruminant fats dominate. Potential use for cooking or consuming food. |
|  |  | S-shaped | 48 | 16±3.3 | 6±1.3 | 44% | 21±42 | 11 55% | 9 45% | 0 0% |  | Smaller pots with the highest lipid preservation rate with a combined use for dairy and ruminant products. |
|  |  | Indet | 192 | 17.5±4.5 | 6±2.4 | 21% | 24±40 | 7 64% | 3 27% | 1 9% | MCK, Mixtures of dairy and ruminant fats | Pots with evidence both for heating and the mixtures of different fat types. Most coherent with food preparation and cooking. |
|  |  |  |  |  |  |  |  |  |  |  |  |  |
|  |  | No lug | 180 | 18±3.2 | 8±2.2 | 18% | 45±27 | 4 57% | 2 29% | 1 14% |  | Pots with the highest median lipid quantities. Most coherent with food consumption. |
|  |  | Two lugs | 164 | 22±3.7 | 8±2.0 | 20% | 23±33 | 10 50% | 9 45% | 1 5% | MCK, Mixtures of dairy and ruminant fats | Big pots with evidence for heating yielding mixtures of dairy and ruminant fats. Most probably used for cooking, handles may aid transport of the vessel when hot. |
|  |  | Four lugs | 95 | 16±3.9 | 6±1.1 | 23% | 24±44 | 12 57% | 8 38% | 1 5% | Mixtures of dairy and ruminant fats | Shape specialised in churning or similar activities containing high quantities of dairy products. |
| Open | Bowl | Oval | 54 | NC | 7±2.2 | 41% | 20±17 | 16 76% | 5 24% | 0 | Mixtures of dairy and ruminant fats | Oval shaped form with a high lipid reservation rate. Intensive use for the preparation and consumption of dairy products. |
|  |  | Hemispheric | 13 | 15±3.2 | 7±1.0 | *8% | NC | NC | NC | NC | Wax Esters | This shape presents an absence of fat and yields low lipid preservation rates but, when positive, wax esters were detected potentially suggesting being subjected to waterproofing. |
|  |  | Collared | 10 | 15±1.7 | 5±0.7 | *0% | NC | NC | NC | NC |  | Absence of fat suggests this shape was not involved in food preparation (although the sample size is small). |
|  |  | S-Shaped | 56 | 18±2.7 | 6±1.1 | 32% | 14±48 | 12 75% | 4 25% | 0 | Mixtures of dairy and ruminant fats | Medium to large bowls mainly used to the prepare and transform dairy products. |
|  | Cup | | 28 | 12±1.6 | 5±0.9 | 32% | 25±70 | 5 62% | 3 38% | 0 |  | As in miniature vessels, the relatively high quantity of dairy fats in cups suggests they might have been used to consume milk or dairy products. |
|  | Dish | | 4 | 15±2.1 | 7±3.5 | *0% | NC | NC | NC | NC |  | The absence of lipids suggests this shape was not involved in food preparation but only 4 cases studied. |
| Special forms | Miniature vessel | | 1 | NC | 4 | NC | 103 | 1 | 0 | 0 |  | The high amounts of dairy fats detected in this vessel suggests a tiny vessel for milk or dairy products. |
|  | Lid | | 12 | 13.5±4.1 | 9±2.5 | *8% | *15.2 | 0 | 0 | 1 |  | Detection of fats (non-ruminant) in lids could result from exposure to the contents of the pots while cooking. |
|  | Box | | 6 | NC | 7.5±2.1 | *0% | NC | NC | NC | NC |  | The absence of lipids suggests that square vessels and boxes were not typically used to contain or prepare food. |
