# Supplementary material for: From bowls to pots: The dairying revolution in Northwest Turkey, a view from Barcın Höyük, 6600 to 6000 BCE
Source: PLoS One. 2024 May 9;19(5):e0302788. doi: 10.1371/journal.pone.0302788 (PMC11081328; doi:10.1371/journal.pone.0302788)
Supplement: S1 Table — (DOCX) [file pone.0302788.s002.docx]

**Supplementary 2: Dataset of vessel characteristics and biomolecular and isotopic results**

1: Summary table of vessel context and characteristics from samples yielding sufficient quantities of lipids

| Lab number | Layer | PHASE | Aperture | Category | Type | Lugs | Sherd | Part | Estimated diameter (cm) | Thickness (mm) |
| --- | --- | --- | --- | --- | --- | --- | --- | --- | --- | --- |
| 15234 | VIe | 1 | Closed | Pot | Pot indet. | None preserved | Base | Lower | - | 9 |
| 15246 | VIe | 1 | Closed | Pot | Pot indet. | None preserved | Base | Lower | - | 9 |
| *15248* | VId1 | 1 | Indet | indet. | indet. | - | Body indet. | Indet | - | 8 |
| 16815 | VIe | 1 | Closed | Pot | Pot indet. | None preserved | Body indet. | Indet | - | 10 |
| 16818 | VIe | 1 | Closed | Pot | Holemouth | None preserved | Rim | Upper | 16 | 8 |
| *24052* | VId1 | 1 | Indet | indet. | indet. | - | Body indet. | Indet | - | 6 |
| *24054* | VId1 | 1 | Indet | indet. | indet. | - | Base | Lower | - | 12 |
| *24056* | VId1 | 1 | Indet | indet. | indet. | - | Base | Lower | - | 12 |
| 26437 | VId1 | 1 | Closed | Pot | Pot indet. | 2-lug | Handle | Upper | - | 6 |
| 26503 | VIe | 1 | Closed | Pot | Pot indet. | None preserved | Body near base | Lower | - | 8 |
| *30601* | VId1 | 1 | Indet | indet. | indet. | - | Body indet. | Indet | - | 9 |
| 30608 | VId1 | 1 | Closed | Pot | Pot indet. | None preserved | Body near base | Lower | - | 9 |
| 30611 | VId1 | 1 | Open | Bowl | Oval | - | Base | Lower | - | 7 |
| 30612 | VId1 | 1 | Open | Bowl | Oval | - | Base | Lower | - | 7 |
| 30629 | VIe | 1 | Closed | Pot | Holemouth | 2-lug | Rim + lug | Upper | 19 | 9 |
| 30638 | VId1 | 1 | Closed | Pot | Holemouth | None preserved | Body indet. | Indet | - | 11 |
| *30642* | VId1 | 1 | Indet | indet. | indet. | - | Body indet. | Indet | - | 7 |
| 30644 | VId1 | 1 | Open | Bowl | Oval | - | Rim | Upper | - | 8 |
| 30647 | VId1 | 1 | Indet | indet. | indet. | - | Body near base | Lower | - | 10 |
| *32609* | VId1 | 1 | Closed | Pot | Pot indet. | None preserved | Rim | Upper | - | 6 |
| 32611 | VId1 | 1 | Indet | indet. | indet. | - | Base | Lower | - | 11 |
| 32633 | VId1 | 1 | Closed | Pot | Holemouth | None preserved | Rim | Upper | 16 | 8 |
| *32634* | VId1 | 1 | Indet | indet. | indet. | - | Body indet. | Indet | - | 8 |
| *32639* | VId1 | 1 | Indet | indet. | indet. | - | Body indet. | Indet | - | 8 |
| 34755 | VIe | 1 | Closed | Pot | Pot indet. | None preserved | Body indet. | Indet | - | 14 |
| 34765 | VIe | 1 | Closed | Pot | Pot indet. | None preserved | Rim | Upper | - | 7 |
| *34769* | VId1 | 1 | Indet | indet. | indet. | - | Body indet. | Indet | - | 12 |
| 34784 | VId1 | 1 | Closed | Pot | Pot indet. | 2-lug | Handle | Upper | - | 8 |
| 37475 | VId1 | 1 | Open | Bowl | Hemispheric | - | Rim | Upper | 14 | 7 |
| 37479 | VId1 | 1 | Closed | Pot | Pot indet. | 2-lug | Handle | Upper | - | 12 |
| 37482 | VId1 | 1 | Open | Bowl | Oval | - | Handle | Upper | - | 5 |
| 37496 | VId1 | 1 | Closed | Pot | Holemouth | None preserved | Rim | Upper | 19 | 7 |
| *37631* | VId1 | 1 | Indet | indet. | indet. | - | Body indet. | Indet | - | 11 |
| 37641 | VId1 | 1 | Closed | Pot | Pot indet. | None preserved | Body indet. | Indet | - | 12 |
| 37647 | VId1 | 1 | Closed | Pot | Holemouth | None preserved | Rim | Upper | 17 | 9 |
| 37648 | VId1 | 1 | Special form | Lid | Lid | - | Rim | Upper | - | 10 |
| 37649 | VId1 | 1 | Closed | Pot | Pot indet. | None preserved | Body indet. | Indet | - | 13 |
| 37650 | VId1 | 1 | Open | Bowl | Oval | - | Rim | Upper | - | 9 |
| 37678 | VId1 | 1 | Closed | Pot | Pot indet. | None preserved | Body indet. | Indet | - | 7 |
| 42702 | VId1 | 1 | Open | Bowl | Oval | - | Rim | Upper | - | 5 |
| 42706 | VId1 | 1 | Indet | indet. | indet. | - | Body indet. | Indet | - | 9 |
| 42708 | VIe | 1 | Closed | Pot | Holemouth | None preserved | Rim | Upper | 15 | 7 |
| 42709 | VIe | 1 | Closed | Pot | Pot indet. | None preserved | Body indet. | Indet | - | 7 |
| 42713 | VIe | 1 | Closed | Pot | Holemouth | None preserved | Rim | Upper | 18 | 11 |
| 42715 | VId1 | 1 | Closed | Pot | Pot indet. | None preserved | Base | Lower | - | 5 |
| 42722 | VId1 | 1 | Closed | Pot | Pot indet. | None preserved | Base | Lower | - | 15 |
| 43660 | VId1 | 1 | Closed | Pot | Holemouth | None preserved | Rim | Upper | 16 | 5 |
| 43663 | VIe | 1 | Closed | Pot | Holemouth | None preserved | Rim | Upper | 20 | 10 |
| 43666 | VIe | 1 | Closed | Pot | Pot indet. | None preserved | Base | Lower | - | 15 |
| 43676 | VId1 | 1 | Indet | indet. | indet. | - | Body indet. | Indet | - | 9 |
| 43697 | VId1 | 1 | Closed | Pot | Holemouth | None preserved | Rim | Upper | - | 8 |
| 47318 | VId1 | 1 | Indet | indet. | indet. | - | Base | Lower | - | 13 |
| 9949 | VId3 | 2 | Indet | indet. | indet. | - | Body indet. | Indet | - | 6 |
| 10138 | VIc | 2 | Open | Bowl | S-shaped | - | Rim | Upper | 21 | 5 |
| 10143 | VIc | 2 | Open | Bowl | S-shaped | - | Rim | Upper | 22 | 6 |
| 10166 | VId2 | 2 | Open | Bowl | Oval | - | Rim | Upper | - | 5 |
| 10167 | VId2 | 2 | Open | Bowl | Oval | - | Rim | Upper | - | 5 |
| 10174 | VId2 | 2 | Closed | Pot | Pot indet. | 2-lug | Lug (solid) 802 | Upper | 22 | 7 |
| 10175 | VId2 | 2 | Open | Bowl | Oval | - | Rim | Upper | - | 7 |
| 11856 | VId2 | 2 | Open | Bowl | Oval | - | Handle | Upper | - | 7 |
| 13401 | VId2 | 2 | Open | Bowl | Oval | - | Rim + handle | Upper | - | 6 |
| 13410 | VId2 | 2 | Indet | indet. | indet. | - | Base | Lower | - | 8 |
| 13464 | VIc | 2 | Open | Bowl | S-shaped | - | Rim | Upper | 24 | 7 |
| 13478 | VIc | 2 | Closed | Pot | Collared neck | 2-lug | Rim | Upper | 16 | 8 |
| 15201 | VIc | 2 | Open | Bowl | S-shaped | - | Rim | Upper | 16 | 7 |
| 15204 | VIc | 2 | Open | Bowl | S-shaped | - | Rim | Upper | 18 | 6 |
| 16819 | VIc | 2 | Open | Bowl | Oval | - | Rim | Upper | - | 7 |
| 16823 | VId2 | 2 | Open | Bowl | Oval | - | Rim | Upper | - | 10 |
| 16840 | VIc | 2 | Closed | Pot | S-shaped | 2-lug | Rim | Upper | 16 | 7 |
| 19930 | VId3 | 2 | Open | Bowl | Hemispheric | - | Rim | Upper | 22 | 7 |
| 19938 | VIc | 2 | Open | Cup | Cup | - | Rim | Upper | 11 | 3 |
| 19948 | VId2 | 2 | Indet | indet. | indet. | - | Body indet. | Indet | - | 8 |
| *24071* | VId2 | 2 | Indet | indet. | indet. | - | Base | Lower | - | 11 |
| *24074* | VId2 | 2 | Indet | indet. | indet. | - | Base | Lower | - | 11 |
| 24076 | VId2 | 2 | Open | Bowl | Oval | - | Base | Lower | - | 7 |
| 24090 | VIc | 2 | Open | Cup | Cup | - | Body indet. | Indet | - | 6 |
| 26392 | VId2 | 2 | Closed | Pot | Pot indet. | None preserved | Rim | Upper | 12 | 5 |
| 26428 | VId2 | 2 | Indet | indet. | indet. | - | Body indet. | Indet | - | 9 |
| 26439 | VId2 | 2 | Open | Bowl | Oval | - | Rim | Upper | - | 5 |
| 26441 | VIc | 2 | Closed | Pot | Pot indet. | 2 or 4 lugs | Handle | Upper | - | 6 |
| 26510 | VIc | 2 | Open | Bowl | S-shaped | - | Body near neck | Upper | - | 4 |
| 26512 | VIc | 2 | Closed | Pot | Collared neck | 2-lug | Body near neck | Upper | - | 7 |
| 30623 | VId2 | 2 | Open | Bowl | Oval | - | Rim | Upper | - | 8 |
| 30650 | VIc | 2 | Open | Cup | Cup | - | Rim | Upper | 13 | 5 |
| 32619 | VId3 | 2 | Open | Bowl | S-shaped | - | Rim | Upper | 19 | 7 |
| 32645 | VId2 | 2 | Open | Bowl | Oval | - | Rim | Upper | - | 7 |
| *34762* | VId2 | 2 | Indet | indet. | indet. | - | Rim | Upper | - | 8 |
| 34789 | VId2 | 2 | Closed | Pot | Pot indet. | None preserved | Body near shoulder | Upper | - | 8 |
| 34797 | VId3 | 2 | Closed | Pot | Collared neck | 2-lug | Rim | Upper | 23 | 8 |
| 37492 | VId3 | 2 | Open | Bowl | S-shaped | - | Rim | Upper | 17 | 9 |
| 37640 | VId3 | 2 | Closed | Pot | Pot indet. | 2-lug | Handle | Upper | - | 8 |
| *37673* | VIc | 2 | Indet | indet. | indet. | - | Body near shoulder | Upper | - | 7 |
| *37674* | VIc | 2 | Closed | Pot | Pot indet. | None preserved | Body indet. | Lower | - | 5 |
| 37724 | VId2 | 2 | Closed | Pot | Pot indet. | 2-lug | Body indet. | Lower | - | 9 |
| 42726 | VId3 | 2 | Closed | Pot | Pot indet. | None preserved | Base | Lower | - | 8 |
| 43661 | VId3 | 2 | Closed | Pot | Collared neck | 2-lug | Rim | Upper | 20 | 8 |
| 43662 | VId2 | 2 | Closed | Pot | Holemouth | None preserved | Rim | Upper | 20 | 7 |
| 43671 | VId2 | 2 | Indet | indet. | indet. | - | Body indet. | Indet | - | 10 |
| 43680 | VId2 | 2 | Indet | indet. | indet. | - | Body indet. | Indet | - | 12 |
| 43686 | VId2 | 2 | Open | Bowl | Oval | - | Rim | Upper | 23 | 7 |
| 43777 | VId2 | 2 | Indet | indet. | indet. | - | Rim | Upper | - | 12 |
| 43787 | VId2 | 2 | Indet | indet. | indet. | - | Rim | Upper | - | 10 |
| *47316* | VId2 | 2 | Closed | Pot | Holemouth | 2-lug | Rim | Upper | - | 7 |
| 2473 | VIb | 3 | Indet | indet. | indet. | - | Body indet. | Indet | - | - |
| 2499 | VIb | 3 | Indet | indet. | indet. | - | Body indet. | Indet | - | - |
| 3164 | VIb | 3 | Indet | indet. | indet. | - | Body indet. | Indet | - | - |
| 3181 | VIb | 3 | Indet | indet. | indet. | - | Base | Lower | - | - |
| 3195 | VIa | 3 | Indet | indet. | indet. | - | Body indet. | Indet | - | 9 |
| 4035 | VIa | 3 | Closed | Pot | Collared neck | 2-lug | Rim | Upper | 16 | 7 |
| 4036 | VIa | 3 | Closed | Pot | Collared neck | 2-lug | Rim | Upper | 18 | 5 |
| 6618 | VIb | 3 | Open | Bowl | Oval | - | Rim | Upper | - | 5 |
| 6619 | VIb | 3 | Closed | Pot | Collared neck | 2-lug | Rim | Upper | 21 | 5 |
| 6625 | VIb | 3 | Closed | Pot | Collared neck | 4-lug | Rim | Upper | 12 | 5 |
| 6628 | VIb | 3 | Closed | Pot | S-shaped | 4-lug | Rim | Upper | 19 | 5 |
| 6630 | VIb | 3 | Open | Bowl | Oval | - | Rim | Upper | - | 7 |
| 6903 | VIb | 3 | Open | Cup | Cup | - | Rim | Upper | 14 | 4 |
| 6905 | VIb | 3 | Open | Bowl | S-shaped | - | Rim | Upper | 17 | 8 |
| 6906 | VIb | 3 | Closed | Pot | Collared neck | 2-lug | Rim | Upper | 19 | 8 |
| 6906 | VIb | 3 | Open | Bowl | S-shaped | - | Rim | Upper | 16 | 5 |
| 6909 | VIa | 3 | Closed | Pot | S-shaped | 2-lug | Rim | Upper | 18 | 7 |
| 6910 | VIb | 3 | Closed | Pot | Pot indet. | 2-lug | Handle | Lower | 18 | 6 |
| 6912 | VIb | 3 | Closed | Pot | Pot indet. | 2-lug | Handle | Upper | 22 | 6 |
| 6913 | VIb | 3 | Closed | Pot | S-shaped | 4-lug | Rim | Upper | 17 | 6 |
| 6914 | VIb | 3 | Open | Cup | Cup | - | Rim | Upper | 12 | 5 |
| 6919 | VIb | 3 | Closed | Pot | Pot indet. | 4-lug | Handle | Upper | 14 | 5 |
| 6920 | VIb | 3 | Open | Bowl | S-shaped | - | Rim | Upper | 16 | 7 |
| 6925 | VIb | 3 | Closed | Pot | Pot indet. | 4-lug | Handle | Upper | 16 | 6 |
| 6933 | VIb | 3 | Open | Bowl | Oval | - | Rim | Upper | - | 8 |
| 6935 | VIb | 3 | Open | Cup | Cup | - | Rim | Upper | 11 | 5 |
| 6935 | VIb | 3 | Closed | Pot | Holemouth | 2-lug | Rim | Upper | 16 | 7 |
| 6935 | VIb | 3 | Closed | Pot | Pot indet. | 4-lug | Handle | Upper | 18 | 6 |
| 6938 | VIa | 3 | Closed | Pot | Pot indet. | 2-lug | Handle | Upper | 18 | 5 |
| 6941 | VIb | 3 | Closed | Pot | S-shaped | 2-lug | Rim | Upper | 15 | 6 |
| 6941 | VIb | 3 | Closed | Pot | S-shaped | 4-lug | Rim | Upper | 15 | 9 |
| 6941 | VIb | 3 | Closed | Pot | Collared neck | 2 or 4 lugs | Rim | Upper | 19 | 4 |
| 6947 | VIa | 3 | Closed | Pot | S-shaped | 4-lug | Rim | Upper | 19 | 7 |
| 6948 | VIb | 3 | Open | Cup | Cup | - | Rim | Upper | 14 | 6 |
| 6949 | VIb | 3 | Closed | Pot | S-shaped | 2 or 4 lugs | Rim | Upper | 18 | 6 |
| 6950 | VIa | 3 | Closed | Pot | S-shaped | 4-lug | Rim | Upper | 16 | 6 |
| 6952.1 | VIa | 3 | Closed | Pot | S-shaped | 4-lug | Rim | Upper | 18 | 3 |
| 6952.2 | VIa | 3 | Closed | Pot | S-shaped | 4-lug | Rim | Upper | 16 | 6 |
| 6952.3 | VIa | 3 | Closed | Pot | Pot indet. | 4-lug | Handle | Upper | 26 | 6 |
| 6956 | VIb | 3 | Closed | Pot | S-shaped | 4-lug | Rim | Upper | 12 | 8 |
| 6959 | VIb | 3 | Closed | Pot | Pot indet. | 2-lug | Handle | Upper | 19 | 5 |
| 6960 | VIb | 3 | Closed | Pot | S-shaped | 2-lug | Rim | Upper | 20 | 7 |
| 10105 | VIb | 3 | Closed | Pot | S-shaped | 4-lug | Rim | Upper | 12 | 6 |
| 10112 | VIa | 3 | Closed | Pot | S-shaped | 2-lug | Rim | Upper | 22 | 5 |
| 10115 | VIa | 3 | Closed | Pot | Pot indet. | 4-lug | Handle | Upper | 17 | 7 |
| 10128 | VIb | 3 | Closed | Pot | S-shaped | 2 or 4 lugs | Rim | Upper | 16 | 8 |
| 10129 | VIa | 3 | Closed | Pot | Pot indet. | 4-lug | Handle | Upper | 23 | 6 |
| 10131 | VIb | 3 | Open | Bowl | S-shaped | - | Rim | Upper | 17 | 6 |
| 10139 | VIa | 3 | Open | Bowl | S-shaped | - | Rim | Upper | 19 | 6 |
| 10148 | VIb | 3 | Closed | Pot | S-shaped | 4-lug | Rim | Upper | 13 | 5 |
| 10161 | VIb | 3 | Closed | Pot | S-shaped | 4-lug | Rim | Upper | 13 | 5 |
| 13404 | VIa | 3 | Open | Bowl | S-shaped | - | Rim | Upper | - | 7 |
| 13406 | VIb | 3 | Open | Bowl | S-shaped | - | Rim | Upper | 15 | 5 |
| 13431 | VIb | 3 | Closed | Pot | S-shaped | 4-lug | Rim | Upper | 11 | 5 |
| 13435 | VIa | 3 | Closed | Pot | Collared neck | 4-lug | Rim | Upper | 15 | 7 |
| 13443 | VIb | 3 | Open | Bowl | Oval | - | Rim | Upper | - | 5 |
| 13444 | VIa | 3 | Closed | Pot | Holemouth | 2-lug | Rim | Upper | 19 | 8 |
| 13449 | VIb | 3 | Open | Bowl | S-shaped | - | Rim | Upper | 19 | 7 |
| 13451 | VIb | 3 | Closed | Pot | S-shaped | 4-lug | Rim | Upper | 11 | 6 |
| 13458 | VIb | 3 | Closed | Pot | Pot indet. | 4-lug | Handle | Upper | - | 8 |
| 13472 | VIa | 3 | Open | Bowl | S-shaped | - | Rim | Upper | 15 | 7 |
| 13473 | VIa | 3 | Closed | Pot | Pot indet. | 2-lug | Handle | Upper | - | 7 |
| 15205 | VIa | 3 | Open | Bowl | Oval | - | Rim | Upper | - | 8 |
| 15237 | VIb | 3 | Closed | Pot | S-shaped | 4-lug | Rim | Upper | 13 | 5 |
| 15244 | VIa | 3 | Open | Cup | Cup | - | Rim | Upper | 14 | 5 |
| 16832 | VIa | 3 | Closed | Pot | Pot indet. | 2-lug | Handle | Upper | 16 | 6 |
| 16833 | VIa | 3 | Open | Bowl | S-shaped | - | Rim | Upper | 16 | 5 |
| 18546 | VIb | 3 | Open | Cup | Cup | - | Rim | Upper | 12 | 4 |
| 19946 | VIb | 3 | Special form | Vessel | Miniature vessel | - | Handle | Upper | - | 4 |
| 26391 | VIb | 3 | Closed | Pot | Pot indet. | None preserved | Base | Lower | - | 11 |

2: Summary table of molecular and isotopic data from vessels with yielding sufficient quantities of lipids. PS: Palmitic to Stearic acid ratio, FFA: Free fatty acids, DAG: Diacylglycerols, TAG: Triacylglycerols, LCK: Mid long chained ketones, WE: Wax esters, TAG M: TAG average carbon number and TAG DF: TAG dispersion factor. The TAG M and TAG DF values were calculated according to [1] Black squares = presence, Dash = absence

| Lab number | Extraction type | Lipid µg·g^-1^ | PS | Main compounds | FFA | DAG | TAG | LCK | WE | TAG M | TAG DF | δ^13^C_16:0_ | δ^13^C_18:0_ | Δ^13^C | Interpretation |
| --- | --- | --- | --- | --- | --- | --- | --- | --- | --- | --- | --- | --- | --- | --- | --- |
| 15234 | Solvent | around 5 | 0.5 | Fatty acids | 1 | 1 | 1 | - | - | 51.46 | 1.17 | -23.9 | -25.9 | -2.0 | Ruminant adipose fat |
| 15246 | Solvent | 17.1 | - | Fatty acids | 1 | - | - | - | - | - | - | - | - | - | Degraded animal fat |
| *15248* | Solvent | around 5 | - | Fatty acids | 1 | - | - | 1 | - | - | - | - | - | - | Heated animal fat |
| 16815 | Solvent | 42.7 | 1.1 | Fatty acids | 1 | - | - | - | - | - | - | -24.8 | -28.6 | -3.6 | Dairy ruminant fat |
| 16818 | Solvent | around 5 | 1.1 | Fatty acids | 1 | - | - | - | - | - | - | -22.0 | -25.7 | -3.7 | Dairy ruminant fat |
| *24052* | Solvent | 10 | 0.8 | Fatty acids | 1 | - | 1 | - | - | 52.20 | 1.07 | -24.2 | -25.7 | -1.4 | Ruminant adipose fat |
| *24054* | Solvent | 16.5 | 0.5 | Fatty acids | 1 | - | 1 | - | - | 52.97 | 0.94 | -26.7 | -28.0 | -1.2 | Ruminant adipose fat |
| *24056* | Solvent | 7.7 | 0.6 | Fatty acids | 1 | - | 1 | - | - | 50.63 | 1.03 | -26.2 | -28.3 | -2.1 | Ruminant adipose fat |
| 26437 | Solvent | around 5 | 1.2 | Fatty acids | 1 | - | 1 | - | - | 49.02 | 1.59 | -27.1 | -29.7 | -2.6 | Ruminant adipose fat |
| 26503 | Solvent | 6.0 | - | Fatty acids | 1 | - | - | - | - | - | - | - | - | - | Degraded animal fat |
| *30601* | Solvent | around 5 | - | Fatty acids | 1 | - | 1 | - | - | - | - | - | - | - | Degraded animal fat |
| 30608 | Solvent | 62.8 | 1.3 | Fatty acids | 1 | - | 1 | - | - | 47.67 | 1.40 | -27.2 | -29.9 | -2.7 | Ruminant adipose fat |
| 30611 | Solvent | around 5 | 1.2 | Fatty acids | 1 | - | 1 | - | - | 46.73 | 1.09 | -28.7 | -32.2 | -3.4 | Dairy ruminant fat |
| 30612 | Solvent | 17.1 | 0.6 | Fatty acids | 1 | - | 1 | - | - | 48.93 | 1.50 | -27.4 | -31.2 | -3.8 | Dairy ruminant fat |
| 30629 | Solvent | 13.6 | 1.2 | Fatty acids | 1 | - | - | - | - | - | - | -25.2 | -30.1 | -4.9 | Dairy ruminant fat |
| 30638 | Solvent | 8.0 | - | Fatty acids | 1 | - | 1 | - | - | - | - | - | - | - | Degraded animal fat |
| *30642* | Solvent | 7.9 | 1.3 | Fatty acids | 1 | - | 1 | - | - | 49.66 | 1.28 | -26.5 | -32.4 | -5.9 | Dairy ruminant fat |
| 30644 | Solvent | 35.2 | 0.4 | Fatty acids | 1 | - | - | - | - | - | - | -27.4 | -29.8 | -2.4 | Ruminant adipose fat |
| 30647 | Solvent | around 5 | 1.0 | Fatty acids | 1 | - | 1 | - | - | 50.22 | 1.54 | -26.7 | -30.9 | -4.2 | Dairy ruminant fat |
| *32609* | Solvent | around 5 | - | Fatty acids | 1 | - | - | 1 | - | - | - | - | - | - | Heated animal fat |
| 32611 | Solvent | 28.6 | 1.0 | Fatty acids | 1 | - | 1 | - | - | 50.26 | 1.85 | -26.5 | -29.4 | -2.9 | Ruminant adipose fat |
| 32633 | Solvent | 6.9 | 0.7 | Fatty acids | 1 | - | - | - | - | - | - | -25.8 | -28.6 | -2.8 | Ruminant adipose fat |
| *32634* | Solvent | around 5 | 1.0 | Fatty acids | 1 | - | - | - | - | - | - | -23.5 | -29.0 | -5.5 | Dairy ruminant fat |
| *32639* | Solvent | around 5 | 0.9 | Fatty acids | 1 | - | - | - | - | - | - | -26.4 | -29.7 | -3.4 | Dairy ruminant fat |
| 34755 | Solvent | around 5 | - | Wax esters | 1 | - | - | - | 1 | - | - | - | - | - | Wax |
| 34765 | Solvent | 15.8 | 0.6 | Fatty acids | 1 | - | - | - | - | - | - | -25.9 | -28.5 | -2.6 | Ruminant adipose fat |
| *34769* | Solvent | around 5 | 1.0 | Fatty acids | 1 | - | 1 | - | - | 48.26 | 1.76 | -26.0 | -31.1 | -5.1 | Dairy ruminant fat |
| 34784 | Solvent | 33 | 0.8 | Fatty acids | 1 | 1 | 1 | - | - | 48.65 | 1.63 | -26.5 | -29.4 | -2.9 | Ruminant adipose fat |
| 37475 | Solvent | around 5 | - | Wax esters | 1 | - | - | - | 1 | - | - | - | - | - | Wax |
| 37479 | Acid | around 5 | - | Fatty acids | 1 | - | - | - | - | - | - | - | - | - | Degraded animal fat |
| 37482 | Acid | 6 | 0.9 | Fatty acids | 1 | - | - | - | - | - | - | -28.9 | -32.7 | -3.8 | Dairy ruminant fat |
| 37496 | Solvent + Acid | 45.9 | 0.5 | Fatty acids | 1 | - | 1 | - | - | 49.77 | 1.70 | -27.3 | -32.8 | -5.4 | Dairy ruminant fat |
| *37631* | Acid | around 5 | 1.7 | Fatty acids | 1 | - | - | - | - | - | - | -27.1 | -31.7 | -4.6 | Dairy ruminant fat |
| 37641 | Acid | 5.9 | 1.5 | Fatty acids | 1 | - | - | - | - | - | - | -26.7 | -31.1 | -4.4 | Dairy ruminant fat |
| 37647 | Acid | around 5 | - | Fatty acids | 1 | - | - | - | - | - | - | - | - | - | Degraded animal fat |
| 37648 | Solvent + Acid | 15.2 | 1.6 | Fatty acids | 1 | - | - | - | - | - | - | -28.6 | -27.5 | 1.2 | Non-Ruminant fat |
| 37649 | Solvent + Acid | 49.5 | 0.9 | Fatty acids | 1 | 1 | 1 | - | - | 50.06 | 1.54 | -25.5 | -29.1 | -3.6 | Dairy ruminant fat |
| 37650 | Acid | 13.5 | - | Fatty acids | 1 | - | - | - | - | - | - | - | - | - | Degraded animal fat |
| 37678 | Solvent + Acid | 18.2 | 1.8 | Fatty acids | 1 | - | 1 | - | - | 50.05 | 1.08 | -25.6 | -31.5 | -5.9 | Dairy ruminant fat |
| 42702 | Solvent + Acid | 27 | 1.0 | Fatty acids | 1 | - | - | - | - | - | - | -30.0 | -33.0 | -3.0 | Dairy ruminant fat |
| 42706 | Acid | 11 | - | Fatty acids | 1 | - | - | - | - | - | - | - | - | - | Degraded animal fat |
| 42708 | Solvent + Acid | 67.7 | 2.4 | Fatty acids | 1 | - | - | - | - | - | - | -25.1 | -30.1 | -5.0 | Dairy ruminant fat |
| 42709 | Acid | around 5 | 1.2 | Fatty acids | 1 | - | - | - | - | - | - | -26.6 | -30.7 | -4.1 | Dairy ruminant fat |
| 42713 | Acid | around 5 | - | Fatty acids | 1 | - | - | - | - | - | - | - | - | - | Degraded animal fat |
| 42715 | Acid | 22 | 0.3 | Fatty acids | 1 | - | - | - | - | - | - | -31.3 | -31.0 | 0.2 | Non-Ruminant fat |
| 42722 | Acid | 11.3 | 2.2 | Fatty acids | 1 | - | - | - | - | - | - | -27.5 | -33.3 | -6.0 | Dairy ruminant fat |
| 43660 | Solvent + Acid | 52.7 | 1.8 | Fatty acids | 1 | - | - | - | - | - | - | -28.4 | -32.5 | -4.1 | Dairy ruminant fat |
| 43663 | Solvent + Acid | 74 | 1.0 | Fatty acids | 1 | - | - | - | - | - | - | -27.8 | -29.3 | -1.5 | Ruminant adipose fat |
| 43666 | Acid | 6.4 | 1.1 | Fatty acids | 1 | - | - | - | - | - | - | -28.3 | -30.6 | -2.3 | Ruminant adipose fat |
| 43676 | Acid | 12.8 | - | Fatty acids | 1 | - | - | - | - | - | - | - | - | - | Degraded animal fat |
| 43697 | Acid | 20 | 0.8 | Fatty acids | 1 | - | - | - | - | - | - | -28.8 | -32.4 | -3.5 | Dairy ruminant fat |
| 47318 | Solvent + Acid | 43 | - | Fatty acids | 1 | - | - | - | - | - | - | -30.5 | -33.1 | -2.7 | Ruminant adipose fat |
| 9949 | Solvent | around 5 | - | Fatty acids | 1 | - | - | - | - | - | - | - | - | - | Degraded animal fat |
| 10138 | Solvent | 139.1 | 0.6 | Fatty acids | 1 | 1 | 1 | - | - | 50.28 | 1.76 | -25.4 | -27.2 | -1.8 | Ruminant adipose fat |
| 10143 | Solvent | 58.2 | 1.2 | Fatty acids | 1 | 1 | 1 | - | - | 48.12 | 1.52 | -29.2 | -32.5 | -3.2 | Dairy ruminant fat |
| 10166 | Solvent | around 5 | 0.5 | Fatty acids | 1 | - | 1 | - | - | 50.80 | 1.45 | -23.4 | -28.5 | -5.2 | Dairy ruminant fat |
| 10167 | Solvent | 17.7 | 0.8 | Fatty acids | 1 | - | 1 | - | - | 50.21 | 1.57 | -25.7 | -29.0 | -3.3 | Dairy ruminant fat |
| 10174 | Solvent | 6.1 | 1.0 | Fatty acids | 1 | - | - | 1 | - | - | - | -26.1 | -30.3 | -4.2 | Heated dairy ruminant fat |
| 10175 | Solvent | 28.9 | 0.9 | Fatty acids | 1 | - | 1 | - | - | 51.01 | 1.50 | -25.1 | -29.8 | -4.6 | Dairy ruminant fat |
| 11856 | Solvent | 22.7 | 1.0 | Fatty acids | 1 | - | 1 | - | - | 50.03 | 1.62 | -25.6 | -30.6 | -5.0 | Dairy ruminant fat |
| 13401 | Solvent | 50.9 | 0.9 | Fatty acids | 1 | - | - | - | - | - | - | -25.0 | -28.9 | -3.8 | Dairy ruminant fat |
| 13410 | Solvent | around 5 | 4.0 | Fatty acids | 1 | - | - | - | - | - | - | -21.5 | -27.9 | -6.4 | Mix of dairy and plant? |
| 13464 | Solvent | 28.1 | 0.6 | Fatty acids | 1 | 1 | 1 | - | - | 50.72 | 1.44 | -25.5 | -27.6 | -2.0 | Ruminant adipose fat |
| 13478 | Solvent | 117.4 | 0.5 | Fatty acids | 1 | - | 1 | - | - | 51.11 | 1.51 | -32.1 | -33.5 | -1.4 | Ruminant adipose fat |
| 15201 | Solvent | around 5 | 0.7 | Fatty acids | 1 | - | - | - | - | - | - | -20.5 | -24.9 | -4.4 | Dairy ruminant fat |
| 15204 | Solvent | around 5 | 0.7 | Fatty acids | 1 | - | 1 | - | - | 51.66 | 1.39 | -25.4 | -26.9 | -1.5 | Ruminant adipose fat |
| 16819 | Solvent | 48.8 | 0.4 | Fatty acids | 1 | - | 1 | - | - | 48.34 | 1.32 | -27.3 | -29.3 | -2.0 | Ruminant adipose fat |
| 16823 | Solvent | 21.2 | - | Fatty acids | 1 | 1 | 1 | - | - | 49.37 | 1.73 | -25.6 | -26.3 | -0.6 | Ruminant adipose fat |
| 16840 | Solvent | 77 | 0.8 | Fatty acids | 1 | 1 | 1 | - | - | 49.35 | 1.49 | -26.0 | -29.8 | -3.8 | Dairy ruminant fat |
| 19930 | Solvent | around 5 | - | Wax esters | 1 | - | - | - | 1 | - | - | - | - | - | Wax |
| 19938 | Solvent | 223 | 1.0 | Fatty acids | 1 | - | - | - | - | - | - | -22.3 | -28.0 | -5.7 | Dairy ruminant fat |
| 19948 | Solvent | 10.6 | 0.9 | Fatty acids | 1 | - | - | - | - | - | - | -27.6 | -29.6 | -2.0 | Ruminant adipose fat |
| *24071* | Solvent | 10.9 | 0.5 | Fatty acids | 1 | 1 | 1 | - | - | 50.56 | 1.38 | -26.5 | -27.9 | -1.4 | Ruminant adipose fat |
| *24074* | Solvent | 17.1 | 0.4 | Fatty acids | 1 | 1 | 1 | - | - | 51.86 | 1.26 | -28.0 | -31.0 | -3.0 | Ruminant adipose fat |
| 24076 | Solvent | 21.6 | 0.7 | Fatty acids | 1 | - | - | - | - | - | - | -28.9 | -32.1 | -3.1 | Dairy ruminant fat |
| 24090 | Solvent | 8.6 | - | Fatty acids | 1 | - | - | - | - | - | - | - | - | - | Degraded animal fat |
| 26392 | Solvent | around 5 | - | Fatty acids | 1 | - | - | - | - | - | - | - | - | - | Degraded animal fat |
| 26428 | Solvent | 158 | 1.3 | Fatty acids | 1 | - | 1 | - | - | 49.19 | 1.53 | -25.1 | -30.4 | -5.3 | Dairy ruminant fat |
| 26439 | Solvent | 17 | 1.4 | Fatty acids | 1 | - | 1 | - | - | 47.86 | 1.81 | -26.2 | -29.6 | -3.4 | Dairy ruminant fat |
| 26441 | Solvent | 38 | 0.8 | Fatty acids | 1 | 1 | 1 | - | - | 48.10 | 1.04 | -27.6 | -28.4 | -0.7 | Ruminant adipose fat |
| 26510 | Solvent | around 5 | - | Fatty acids | 1 | - | - | - | - | - | - | - | - | - | Degraded animal fat |
| 26512 | Solvent | 6.2 | 1.8 | Fatty acids | 1 | - | 1 | - | - | 50.75 | 0.91 | -28.8 | -29.5 | -0.6 | Ruminant adipose fat |
| 30623 | Solvent | 15.4 | 1.1 | Fatty acids | 1 | - | 1 | - | - | 51.69 | 1.47 | -26.5 | -29.8 | -3.3 | Dairy ruminant fat |
| 30650 | Solvent | 26 | 1.1 | Fatty acids | 1 | 1 | 1 | - | - | 50.96 | 1.31 | -23.7 | -25.9 | -1.9 | Ruminant adipose fat |
| 32619 | Solvent | 11.5 | 1.0 | Fatty acids | 1 | - | 1 | - | - | 49.43 | 1.59 | -24.0 | -28.4 | -4.4 | Dairy ruminant fat |
| 32645 | Solvent | 17.8 | 0.9 | Fatty acids | 1 | - | - | - | - | - | - | -25.3 | -29.3 | -3.9 | Dairy ruminant fat |
| *34762* | Solvent | 10.8 | - | Wax esters | 1 | - | - | - | 1 | - | - | - | - | - | Wax |
| 34789 | Solvent | 22.2 | 1.0 | Fatty acids | 1 | - | - | - | - | - | - | -28.2 | -32.8 | -4.6 | Dairy ruminant fat |
| 34797 | Acid | around 5 | 1.0 | Fatty acids | 1 | - | - | - | - | - | - | -24.1 | -28.5 | -4.4 | Dairy ruminant fat |
| 37492 | Acid | around 5 | - | Wax esters | 1 | - | - | - | 1 | - | - | - | - | - | Wax |
| 37640 | Acid | 7.29 | - | Fatty acids | 1 | - | - | - | - | - | - | - | - | - | Degraded animal fat |
| *37673* | Acid | 15 | 0.9 | Fatty acids | 1 | - | - | - | - | - | - | -28.9 | -32.2 | -3.3 | Dairy ruminant fat |
| *37674* | Solvent | 24 | 0.6 | Fatty acids | 1 | - | - | - | - | - | - | -28.8 | -30.9 | -2.1 | Ruminant adipose fat |
| 37724 | Acid | 10 | 0.6 | Fatty acids | 1 | - | - | - | - | - | - | -30.3 | -31.7 | -1.4 | Ruminant adipose fat |
| 42726 | Acid | 49.5 | 2.6 | Fatty acids | 1 | - | - | - | - | - | - | -28.1 | -28.6 | -0.5 | Ruminant adipose fat |
| 43661 | Acid | 21.3 | 2.1 | Fatty acids | 1 | - | - | - | - | - | - | -27.7 | -29.7 | -2.0 | Ruminant adipose fat |
| 43662 | Solvent + Acid | 44.8 | 0.8 | Fatty acids | 1 | - | - | - | - | - | - | -28.8 | -28.4 | 0.4 | Non-Ruminant fat |
| 43671 | Acid | around 5 | - | Fatty acids | 1 | - | - | - | - | - | - | - | - | - | Degraded animal fat |
| 43680 | Acid | around 5 | - | Fatty acids | 1 | - | - | - | - | - | - | - | - | - | Degraded animal fat |
| 43686 | Acid | 19.9 | - | Fatty acids | 1 | - | - | - | - | - | - | - | - | - | Degraded animal fat |
| 43777 | Acid | 11 | - | Fatty acids | 1 | - | - | - | - | - | - | - | - | - | Degraded animal fat |
| 43787 | Acid | around 5 | - | Fatty acids | 1 | - | - | - | - | - | - | - | - | - | Degraded animal fat |
| *47316* | Solvent + Acid | 42.3 | - | Fatty acids | 1 | - | - | - | - | - | - | -29.5 | -32.7 | -3.2 | Dairy ruminant fat |
| 2473 | Solvent | 162 | 0.9 | Fatty acids | 1 | - | 1 | - | - | 49.71 | 1.69 | -20.6 | -24.7 | -4.1 | Dairy ruminant fat |
| 2499 | Solvent | 30.2 | 1.6 | Fatty acids | 1 | - | - | - | - | - | - | -25.5 | -29.1 | -3.6 | Dairy ruminant fat |
| 3164 | Solvent | 202 | 2.1 | Fatty acids | 1 | - | 1 | - | - | 48.86 | 1.94 | -26.2 | -30.4 | -4.2 | Dairy ruminant fat |
| 3181 | Solvent | around 5 | - | Wax esters | 1 | - | - | - | 1 | - | - | - | - | - | Wax |
| 3195 | Solvent | 303 | 0.7 | Fatty acids | 1 | - | 1 | - | - | 48.77 | 1.87 | -22.7 | -26.9 | -4.2 | Dairy ruminant fat |
| 4035 | Solvent | 74.7 | 0.5 | Fatty acids | 1 | - | - | - | - | - | - | -26.5 | -29.1 | -2.6 | Ruminant adipose fat |
| 4036 | Solvent | 26.4 | 0.6 | Fatty acids | 1 | 1 | 1 | - | - | 51.82 | 1.29 | -25.8 | -27.3 | -2.4 | Ruminant adipose fat |
| 6618 | Solvent | 11 | 1.3 | Fatty acids | 1 | - | - | - | - | - | - | -26.9 | -29.9 | -3.0 | Ruminant adipose fat |
| 6619 | Solvent | 18.9 | 0.6 | Fatty acids | 1 | - | - | - | - | - | - | -26.3 | -28.4 | -2.1 | Ruminant adipose fat |
| 6625 | Solvent | 47.2 | 0.9 | Fatty acids | 1 | - | 1 | - | - | 48.83 | 1.65 | -26.0 | -31.4 | -5.4 | Dairy ruminant fat |
| 6628 | Solvent | 15.6 | 0.8 | Fatty acids | 1 | - | - | - | - | - | - | -29.9 | -32.7 | -2.8 | Ruminant adipose fat |
| 6630 | Solvent | 18.1 | 1.5 | Fatty acids | 1 | - | - | - | - | - | - | -22.5 | -28.0 | -5.5 | Dairy ruminant fat |
| 6903 | Solvent | 67.8 | 1.0 | Fatty acids | 1 | - | - | - | - | - | - | -24.4 | -27.2 | -2.8 | Ruminant adipose fat |
| 6905 | Solvent | around 5 | 0.7 | Fatty acids | 1 | - | - | - | - | - | - | -26.5 | -29.9 | -3.4 | Dairy ruminant fat |
| 6906 | Solvent | 16.5 | 0.5 | Fatty acids | 1 | - | - | - | - | - | - | -26.2 | -32.0 | -5.8 | Dairy ruminant fat |
| 6906 | Solvent | 22 | 0.9 | Fatty acids | 1 | - | - | - | - | - | - | -29.7 | -32.8 | -3.1 | Dairy ruminant fat |
| 6909 | Solvent | around 5 | 1.5 | Fatty acids | 1 | - | - | - | - | - | - | -28.1 | -32.6 | -3.6 | Dairy ruminant fat |
| 6910 | Solvent | 89.4 | 1.0 | Fatty acids | 1 | - | - | - | - | - | - | -24.3 | -28.6 | -4.5 | Dairy ruminant fat |
| 6912 | Solvent | around 5 | - | Fatty acids | 1 | - | - | - | - | - | - | - | - | - | Degraded animal fat |
| 6913 | Solvent | 21.5 | 0.9 | Fatty acids | 1 | - | - | - | - | - | - | -25.4 | -28.2 | -2.8 | Ruminant adipose fat |
| 6914 | Solvent | 25.2 | 1.2 | Fatty acids | 1 | - | - | - | - | - | - | -25.1 | -28.7 | -3.6 | Dairy ruminant fat |
| 6919 | Solvent | 118 | 1.1 | Fatty acids | 1 | - | - | - | - | - | - | -26.3 | -30.3 | -4.0 | Dairy ruminant fat |
| 6920 | Solvent | 17.5 | 1.0 | Fatty acids | 1 | - | - | - | - | - | - | -26.2 | -26.3 | -0.1 | Ruminant adipose fat |
| 6925 | Solvent | 10.5 | 0.9 | Fatty acids | 1 | - | - | - | - | - | - | -21.7 | -26.6 | -4.9 | Dairy ruminant fat |
| 6933 | Solvent | 50.9 | 1.0 | Fatty acids | 1 | - | - | - | - | - | - | -22.8 | -27.2 | -4.3 | Dairy ruminant fat |
| 6935 | Solvent | 5.6 | 1.3 | Fatty acids | 1 | - | - | - | - | - | - | -27.2 | -31.6 | -4.3 | Dairy ruminant fat |
| 6935 | Solvent | 11 | 1.3 | Fatty acids | 1 | - | - | - | - | - | - | -20.0 | -23.0 | -3.0 | Dairy ruminant fat |
| 6935 | Solvent | 12.3 | 0.8 | Fatty acids | 1 | - | - | - | - | - | - | -25.4 | -27.8 | -2.4 | Ruminant adipose fat |
| 6938 | Solvent | 24.6 | 0.7 | Fatty acids | 1 | - | - | - | - | - | - | -28.5 | -31.1 | -2.5 | Ruminant adipose fat |
| 6941 | Solvent | 24.3 | 0.6 | Fatty acids | 1 | - | - | - | - | - | - | -28.5 | -29.9 | -1.4 | Ruminant adipose fat |
| 6941 | Solvent | 48 | 0.9 | Fatty acids | 1 | - | - | - | - | - | - | -25.0 | -26.3 | -1.3 | Ruminant adipose fat |
| 6941 | Solvent | around 5 | - | Fatty acids | 1 | - | - | - | - | - | - | - | - | - | Degraded animal fat |
| 6947 | Solvent | around 5 | 1.0 | Fatty acids | 1 | - | - | - | - | - | - | -28.9 | -32.3 | -3.4 | Dairy ruminant fat |
| 6948 | Solvent | around 5 | 1.2 | Fatty acids | 1 | - | 1 | - | - | 48.01 | 2.20 | -24.7 | -28.0 | -3.3 | Dairy ruminant fat |
| 6949 | Solvent | 13.5 | 0.9 | Fatty acids | 1 | - | - | - | - | - | - | -24.4 | -28.5 | -4.1 | Dairy ruminant fat |
| 6950 | Solvent | 19.4 | 0.9 | Fatty acids | 1 | - | - | - | - | - | - | -26.1 | -27.6 | -1.5 | Ruminant adipose fat |
| 6952.1 | Solvent | 42 | 1.2 | Fatty acids | 1 | - | - | - | - | - | - | -25.3 | -31.0 | -5.6 | Dairy ruminant fat |
| 6952.2 | Solvent | around 5 | 1.0 | Fatty acids | 1 | - | - | - | - | - | - | -25.3 | -30.67 | -5.36 | Dairy ruminant fat |
| 6952.3 | Solvent | around 5 | 0.8 | Fatty acids | 1 | - | 1 | - | - | - | - | -25.7 | -24.08 | 1.65 | Non-Ruminant fat |
| 6956 | Solvent | 162 | 1.1 | Fatty acids | 1 | - | - | - | - | - | - | -27.0 | -31.58 | -4.52 | Dairy ruminant fat |
| 6959 | Solvent | 68 | 0.5 | Fatty acids | 1 | - | 1 | - | - | - | - | -28.1 | -32.6 | -4.51 | Dairy ruminant fat |
| 6960 | Solvent | 21.4 | 1.0 | Fatty acids | 1 | - | - | - | - | - | - | -26.0 | -26.8 | -0.8 | Ruminant adipose fat |
| 10105 | Solvent | 85.4 | 0.8 | Fatty acids | 1 | - | - | - | - | - | - | -24.4 | -27.63 | -3.18 | Dairy ruminant fat |
| 10112 | Solvent | 14.5 | 1.8 | Fatty acids | 1 | - | - | - | - | - | - | -25.3 | -26.63 | -1.33 | Ruminant adipose fat |
| 10115 | Solvent | around 5 | 0.7 | Fatty acids | 1 | - | 1 | - | - | 48.24 | 1.05 | -20.5 | -23.8 | -3.32 | Dairy ruminant fat |
| 10128 | Solvent | 8.2 | 1.2 | Fatty acids | 1 | 1 | 1 | - | - | 50.18 | 1.14 | -24.6 | -29.0 | -4.36 | Dairy ruminant fat |
| 10129 | Solvent | 24.5 | 0.5 | Fatty acids | 1 | - | - | - | - | - | - | -26.2 | -27.96 | -1.71 | Ruminant adipose fat |
| 10131 | Solvent | 89.4 | 0.8 | Fatty acids | 1 | 1 | 1 | - | - | 49.08 | 1.31 | -27.0 | -30.0 | -3.0 | Dairy ruminant fat |
| 10139 | Solvent | 13.6 | 1.1 | Fatty acids | 1 | - | - | - | - | - | - | -26.0 | -29.79 | -3.71 | Dairy ruminant fat |
| 10148 | Solvent | 11.4 | 2.7 | Fatty acids | 1 | - | - | - | - | - | - | -22.3 | -25.48 | -3.15 | Dairy ruminant fat |
| 10161 | Solvent | 96.6 | 0.8 | Fatty acids | 1 | - | - | - | - | - | - | -24.5 | -29.33 | -4.76 | Dairy ruminant fat |
| 13404 | Solvent | 16.6 | 1.0 | Fatty acids | 1 | - | - | - | - | - | - | -28.1 | -31.25 | -3.1 | Dairy ruminant fat |
| 13406 | Solvent | 155.2 | 1.1 | Fatty acids | 1 | - | - | - | - | - | - | -22.5 | -28.32 | -5.79 | Dairy ruminant fat |
| 13431 | Solvent | 99 | 1.5 | Fatty acids | 1 | - | - | - | - | - | - | -28.8 | -32.06 | -3.26 | Dairy ruminant fat |
| 13435 | Solvent | 40.2 | 1.2 | Fatty acids | 1 | - | 1 | - | - | 49.84 | 1.31 | -27.6 | -31.42 | -3.76 | Dairy ruminant fat |
| 13443 | Solvent | 72.3 | 0.9 | Fatty acids | 1 | - | - | 1 | - | - | - | -23.5 | -28.42 | -4.91 | Heated dairy ruminant fat |
| 13444 | Solvent | 55 | 0.6 | Fatty acids | 1 | 1 | 1 | - | - | 51.47 | 1.35 | -28.8 | -28.9 | -0.08 | Non-Ruminant fat |
| 13449 | Solvent | 11.3 | 1.0 | Fatty acids | 1 | - | - | - | - | - | - | -24.8 | -28.82 | -3.99 | Dairy ruminant fat |
| 13451 | Solvent | 25.3 | 1.1 | Fatty acids | 1 | 1 | 1 | - | - | 49.89 | 1.80 | -21.1 | -23.77 | -2.66 | Ruminant adipose fat |
| 13458 | Solvent | 43.6 | 0.8 | Fatty acids | 1 | 1 | 1 | - | - | 51.02 | 1.54 | -25.4 | -25.96 | -0.49 | Non-Ruminant fat |
| 13472 | Solvent | 5.7 | 1.1 | Fatty acids | 1 | - | - | - | - | - | - | -25.2 | -31.06 | -5.87 | Dairy ruminant fat |
| 13473 | Solvent | 26.9 | 0.8 | Fatty acids | 1 | - | 1 | - | - | 50.29 | 1.02 | -25.1 | -26.36 | -1.21 | Ruminant adipose fat |
| 15205 | Solvent | 42.2 | 0.7 | Fatty acids | 1 | - | 1 | - | - | 52.75 | 1.00 | -23.7 | -25.26 | -1.54 | Ruminant adipose fat |
| 15237 | Solvent | 16.1 | 1.7 | Fatty acids | 1 | - | 1 | - | - | 49.33 | 1.58 | -26.0 | -28.38 | -2.31 | Ruminant adipose fat |
| 15244 | Solvent | 40.3 | 0.8 | Fatty acids | 1 | 1 | 1 | - | - | 49.95 | 1.57 | -24.9 | -28.47 | -3.73 | Dairy ruminant fat |
| 16832 | Solvent | 76 | 0.5 | Fatty acids | 1 | 1 | 1 | - | - | 50.30 | 1.15 | -25.2 | -28.38 | -3.11 | Dairy ruminant fat |
| 16833 | Solvent | around 5 | 0.7 | Fatty acids | 1 | - | 1 | - | - | 48.45 | 1.29 | -27.3 | -31.4 | -4.1 | Dairy ruminant fat |
| 18546 | Solvent | 10 | 1.3 | Fatty acids | 1 | - | 1 | - | - | 49.34 | 1.80 | -23.1 | -25.9 | -2.7 | Ruminant adipose fat |
| 19946 | Solvent | 103 | 1.3 | Fatty acids | 1 | - | - | - | - | - | - | -26.9 | -31.7 | -4.7 | Dairy ruminant fat |
| 26391 | Solvent | 37.4 | 0.6 | Fatty acids | 1 | - | - | - | - | - | - | -23.7 | -28.9 | -5.2 | Dairy ruminant fat |

References:

1. Regert M. Analytical strategies for discriminating archaeological fatty substances from animal origin. Mass Spectrom Rev. 2011;30: 177–220.
